# EccDNA‐Driven VPS41 Amplification Alleviates Genotoxic Stress via Lysosomal KAI1 Degradation

**DOI:** 10.1002/advs.202501934

**Published:** 2025-04-24

**Authors:** Bin Shi, Ping Yang, Huaijin Qiao, Jinchen He, Bin Song, Hao Bai, Fengdi Jiang, Yining Zhang, Qian Li, Tao Yan, Wenlin Tu, Daojiang Yu, Shuyu Zhang

**Affiliations:** ^1^ Laboratory of Radiation Medicine West China School of Basic Medical Sciences & Forensic Medicine Sichuan University Chengdu 610041 China; ^2^ Department of Laboratory Medicine Affiliated Hospital of Zunyi Medical University Zunyi 563006 China; ^3^ The Second Affiliated Hospital of Chengdu Medical College China National Nuclear Corporation 416 Hospital Chengdu 610051 China; ^4^ Medical College of Tibet University Tibet University Lhasa China No. 1 South Lubulinka Road Lhasa 850001 China

**Keywords:** circle‐Seq, extrachromosomal circular DNA, genotoxic stress, radiation‐induced skin injury, vacuolar protein sorting 41 homolog (VPS41)

## Abstract

Genotoxic therapies such as ionizing radiation eliminate cancer cells by inducing extensive DNA damage but often cause normal tissue toxicity, including cutaneous injury. Extrachromosomal circular DNA (eccDNA) refers to circular DNA fragments outside the chromosomal context, with their formation and persistence linked to DNA damage repair and genomic instability. Despite growing recognition of eccDNA in oncogenesis, its role under genotoxic stress in normal tissues remains poorly understood. Here, eccDNA is profiled in irradiated rat skin using Circle‐seq, identifying alterations in eccDNA number and composition. Specifically, radiation induced circle^17:44148731‐48208624^, in which vacuolar protein sorting 41 homolog *(VPS41)* is the sole radiation‐induced amplification gene by semiquantitative PCR and gel electrophoresis. The findings show that eccDNA or VPS41 overexpression reduces radiation‐induced skin injury (RISI) in vitro and in vivo. Proteomic and interaction analyses identified metastasis suppressor kangai‐1 (KAI1) as a VPS41‐interacting partner. Notably, VPS41 overexpression promotes KAI1 lysosomal degradation, protecting against radiation‐induced apoptotic cell death. Peptide array analysis pinpoints the VPS41‐KAI1 interaction through the K263 residue, consistent with AlphaFold prediction. The findings uncover a novel mechanism in which radiation‐induced eccDNA, specifically VPS41, mitigates skin injury by modulating KAI1 degradation. This study highlights the role of eccDNA in cellular defense, providing strategies to enhance tissue resilience to genotoxic stress.

## Graphical abstract

Following ionizing radiation, eccDNA‐mediated VPS41 amplification slightly increases its expression but fails to prevent apoptosis. Introducing exogenous eccDNA or VPS41 enhances VPS41‐KAI1 interaction, promoting lysosomal degradation of KAI1. This process inhibits apoptotic signaling, enhancing cell survival and resistance to radiation‐induced damage. These findings reveal a novel regulatory axis with potential implications for radioprotection strategies.

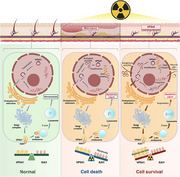

## 1 Introduction

Genotoxic therapies, such as chemotherapy and radiotherapy (RT), remain essential in cancer treatment, primarily by inducing DNA damage to eliminate tumor cells.^[^ ^1^, ^2^ ^]^ However, these therapies are not selective and can also cause significant damage to healthy tissues, depending on factors like treatment type, dosage, and tissue sensitivity. This collateral damage to normal tissues can have serious consequences, affecting both long‐term therapeutic outcomes and patient quality of life.^[^ ^3^, ^4^, ^5^ ^]^ Although researchers have focused on identifying strategies to mitigate these adverse effects, including the use of radioprotective agents, antioxidants, and DNA repair modulators, a key challenge remains in balancing tumor targeting with protection of normal tissues.^[^ ^6^, ^7^, ^8^, ^9^ ^]^

Among the most prevalent side effects of genotoxic therapies, radiation‐induced skin injury (RISI) is a major complication of radiotherapy, occurring in ≈95% of patients.^[^ ^10^, ^11^ ^]^ As the skin is the largest and most exposed organ to radiation damage, radiation‐induced skin injury presents with both acute and chronic manifestations, ranging from erythema and desquamation to fibrosis and ulceration in severe cases.^[^ ^12^, ^13^ ^]^ Beyond localized skin damage, radiation‐induced skin injury can also trigger systemic inflammation, exacerbating fatigue and pain, which may necessitate treatment interruption and ultimately compromise tumor control.^[^ ^14^, ^15^ ^]^ Despite its high prevalence, there is a lack of effective treatments beyond symptomatic management, which primarily relies on emollients, corticosteroids, and protective dressings, while no targeted biological therapies are currently available.^[^ ^16^, ^17^ ^]^ This highlights the urgent need for a deeper understanding of the molecular mechanisms underlying radiation‐induced skin injury to develop novel therapeutic strategies.

Extrachromosomal circular DNA (eccDNA) is DNA fragments that exist outside the chromosomal context, and its formation and persistence are closely linked to DNA repair mechanisms and genomic instability. EccDNA has been identified in a wide range of organisms, from yeast to humans, and is present in both normal and diseased tissues.^[^ ^18^, ^19^, ^20^, ^21^, ^22^ ^]^ Unlike linear chromosomal DNA, eccDNA is highly dynamic and heterogeneous, varying in size, sequence composition, and abundance across different cell types and physiological conditions.^[^ ^19^, ^23^ ^]^ Studies have shown that eccDNA can arise from genomic regions containing gene fragments, repetitive elements, or regulatory sequences, suggesting its involvement in genome plasticity and stress responses.^[^ ^24^, ^25^ ^]^ While eccDNA has been extensively studied in cancer due to its role in oncogene amplification and tumor heterogeneity, emerging evidence suggests that eccDNA may also play functional roles in normal tissues. Notably, recent studies indicate that environmental stressors, including genotoxic agents such as chemotherapy and radiation, can influence eccDNA composition and abundance, raising questions about whether eccDNA may contribute to the cellular response to DNA damage.

Ionizing radiation (IR) is a potent genotoxic stressor that induces a spectrum of DNA lesions, including single‐strand breaks (SSBs), double‐strand breaks (DSBs), and base damage, typically occurring within localized 10–15 base pair segments.^[^ ^26^ ^]^ These lesions trigger a variety of DNA repair mechanisms, some of which may contribute to the generation of eccDNA.^[^ ^19^ ^]^ Recent evidence suggests that eccDNA can arise from DNA fragmentation and subsequent circularization events, potentially as a byproduct of DNA damage repair or as part of an adaptive response to genotoxic stress. Given the ubiquity of eccDNA in various cell types and its apparent sensitivity to environmental stressors, we hypothesize that eccDNA may also contribute to the cellular response to radiation‐induced damage in normal tissues. Investigating how eccDNA dynamics change upon radiation exposure may provide new insights into the mechanisms of genomic stability and stress adaptation.

In this study, we explored the eccDNA landscape in response to ionizing radiation using Circle‐Seq and identified vacuolar protein sorting 41 homolog (VPS41) as a novel radiation‐induced eccDNA gene, with its amplification serving as a defense against radiation‐induced apoptosis. We further revealed a radioprotective mechanism whereby VPS41 reduces KAI1 protein accumulation via lysosomal degradation.

## 2 Results

### 2.1 The Profile Characteristics of eccDNA in Rat Skin Exposed to Ionizing Radiation

**Figure 1:**
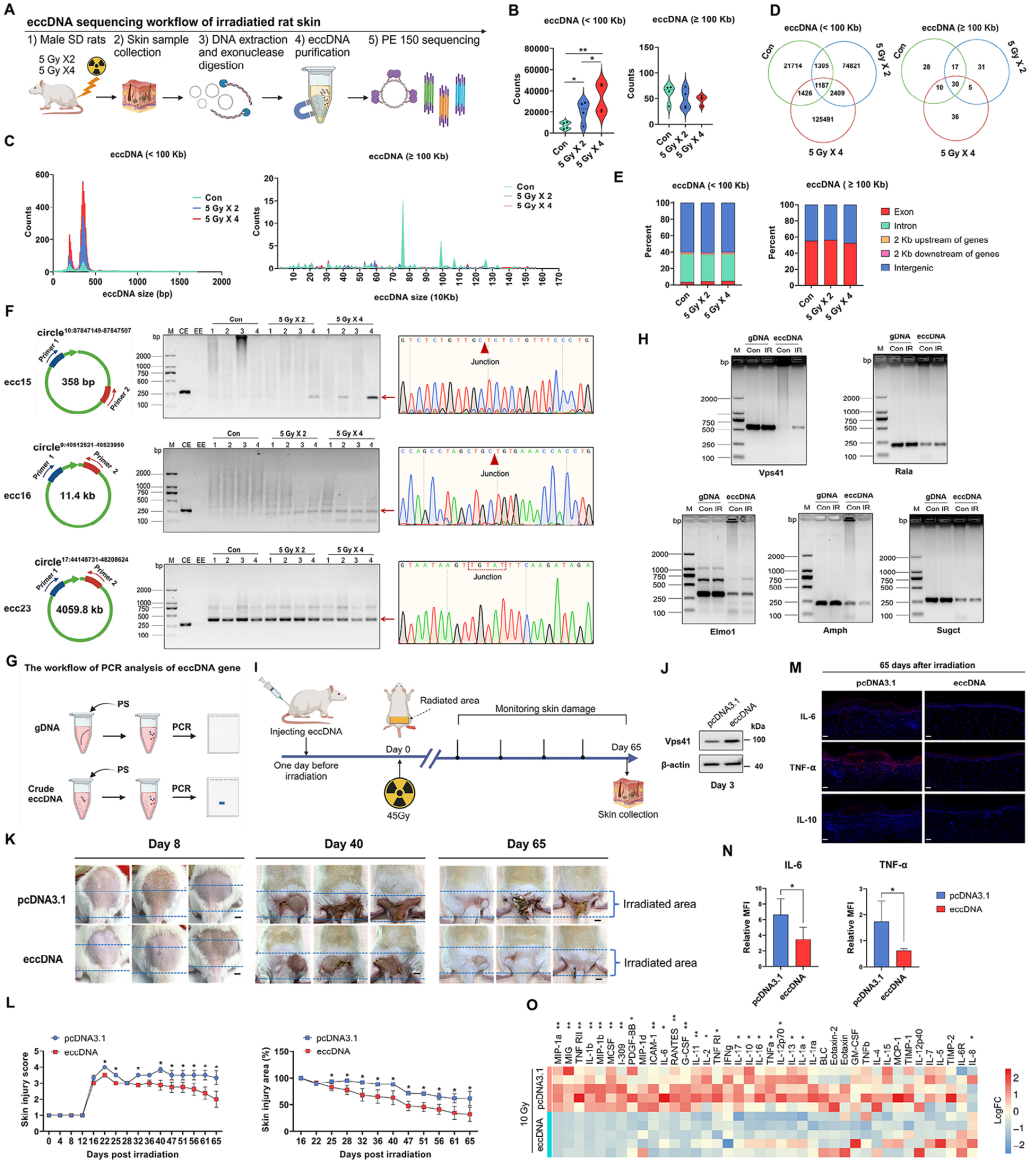
Identification and characterization of eccDNA in rat skin induced by ionizing radiation. A) Flowchart for eccDNA purification and sequencing (*n* = 4 for each group). B) Quantification of unique eccDNA. C) Distribution of unique eccDNA lengths. D) Overlap of eccDNA across sample groups. E) Genomic origins of eccDNA. F) Identification of three eccDNAs using PCR and Sanger sequencing, with circle^17:44148731‐48208624^ (4059.8 Kb) present in all samples (CE: crude eccDNA, EE: exonuclease‐treated eccDNA). G) Flowchart of semiquantitative analysis of PCR by gel electrophoresis on the eccDNA gene. H) PCR detected five genes on circle^17:44148731‐48208624^. I) Flowchart for eccDNA pre‐treatment of rats with radiation‐induced skin injury (*n* = 3 for each group). J) Vps41 protein expression in rat skin 3 days post eccDNA transfection. K) Skin damage photos at 8, 40, and 65 days post‐irradiation in eccDNA‐pre‐treated rats (4 µg injection; scale bar: 1 cm). L) Radiation damage scores and affected areas in eccDNA pre‐treated rats. M,N) Immunofluorescence and analysis of inflammatory factors (IL‐6, IL‐10, TNF‐α) in irradiated skin of eccDNA‐pre‐treated rats (scale bar: 100 µm). O) Inflammatory cytokine array detection in eccDNA‐treated WS1 cells (*n* = 5 per group). *P* values were calculated using different statistical methods based on data type: Mann–Whitney U test for two‐group comparisons, one‐way ANOVA followed by Bonferroni's post hoc test for multi‐group comparisons, and limma's empirical Bayes moderated t‐statistics for high‐throughput expression data. Statistically significant differences are denoted as follows: ^*^ *p* < 0.05, ^**^ *p* < 0.01. Data are presented as mean ± SD (*n* = 3) unless otherwise specified.

To investigate eccDNA profiles in response to ionizing radiation, we developed a rat model of radiation‐induced skin injury by fractional radiation of the gluteal skin (**Figure** **1**A). Two dosages were used: 5 Gy administered twice (5 Gy x 2) and four times (5 Gy x 4) with a 7‐day interval. We applied relatively low radiation doses to investigate the eccDNA response under moderate radiation stress and assess dose‐dependent variations in eccDNA. We omitted rolling circle amplification (RCA) during eccDNA isolation and sequencing to reduce amplification biases^,[^ ^27^, ^28^ ^]^ conducting sequencing on skin tissues from 12 rats (4 rats per treatment group) using Circle‐Seq.

Our analysis showed an a significant increase in unique mean eccDNA counts in irradiated skin compared to controls (mean 7098 in control group, 20 403 in 5 Gy × 2 group, and 33 295 in 5 Gy × 4 group), indicating radiation‐induced elevation in eccDNA (<100 Kb) (Table S1, Supporting Information). Conversely, eccDNA (≥100 Kb) incidence was low, with averages of 61, 50, and 48 in each group, showing a slight decrease post‐irradiation (Figure 1B). The eccDNA length distribution exhibited peaks at 200 bp and 358 bp, consistent with prior studies.^[^ ^29^ ^]^ Increased irradiation doses led to higher proportions of eccDNA at these peaks (Figure 1C). These short eccDNAs may result from the cyclization of small DNA fragments due to radiation.^[^ ^28^ ^]^ Venn diagrams showed some shared eccDNAs across groups, particularly for eccDNA (≥100 Kb), which had over 50% overlap (Figure 1D). Genome composition analysis revealed that over 95% of short eccDNA originates from non‐coding regions, especially intronic and intergenic areas, while long eccDNA primarily comes from exonic regions (Figure 1E). EccDNA from sex chromosomes was lower than from autosomes (Figure S1A, Supporting Information). The cyclization junction sequence was enriched in adenine and thymine (Figure S1B,C, Supporting Information), consistent with prior reports.^[^ ^29^ ^]^ After stringent filtering, we identified 19 eccDNAs (≥ 100 Kb) with split reads >20, containing 150 distinct genes. GO enrichment analysis showed these genes are mainly involved in regulating protein and peptide hydrolases (Figure S1D, Supporting Information).

### 2.2 Irradiation Induces Notable Changes in eccDNA Genes

We detected 40 eccDNAs with elevated read rankings for IGV analysis (data not shown), PCR, and Sanger sequencing verification (Tables S2 and S3, Figures S2B, S3A–H and S4A–H, Supporting Information). Ultimately, we confirmed two small eccDNAs measuring 358 bp and 11.4 Kb (circle^9:40512521‐40523950^ and circle^10:87847149‐87847507^) and one large eccDNA measuring 4059.8 Kb (circle^17:44148731‐48208624^) (Figure 1F, Figure S2A,B,S3A–H, and S4A–H, Supporting Information). The two short eccDNAs predominantly manifested in specific irradiated samples, originating from intergenic or intron regions, indicating the stochastic nature of eccDNA generation. The circle^17:44148731‐48208624^ was derived from the exon region and was consistently present across all samples, including the control group. This eccDNA comprised 13 genes, indicating that these genes may confer significant biological functions to eccDNA. Here, we developed an eccDNA gene detection method by semiquantitative analysis of PCR by gel electrophoresis (Figure 1G), which integrates nucleic acid electrophoresis to confirm whether these genes are present on eccDNA. Our results revealed that 5 of these genes were present on eccDNA, namely *Rala*, *Sugct*, *Vps41*, *Amph* and *Elmo1* (Figure 1H and Figure S5A–E, Supporting Information). *Vps41* was uniquely upregulated on eccDNA in response to irradiation among these genes (Figure 1H and Figure S2B, Supporting Information). Consistently, eccDNA^VPS41^ (ecc23) identified from Circle‐Seq data, also exhibited a significant increase in copy number following irradiation (Figure S2A, Supporting Information). The examination of the UCSC Genome Browser for Human (GRCh38/hg38) revealed that the sequence conservation for genes selected on the eccDNA was significantly greater compared to that of unselected genes (Figure S5F, Supporting Information).

### 2.3 EccDNA Attenuates Radiation‐Induced Skin Injury in Rats

Given the low efficiency of eccDNA transfer into cells, we used an animal model with radiation‐induced skin injury through subcutaneous injection of eccDNA. This approach aims to elucidate the potential role of eccDNA in radiation‐induced skin injury (Figure 1I). The 45 Gy dose used in this model is a well‐established standard for inducing radiation‐induced skin injury in SD rats.^[^ ^30^, ^31^, ^32^ ^]^ Notably, skin damage repair was markedly enhanced in rats receiving eccDNA treatment compared to the control group, accompanied by a significant upregulation of Vps41 expression (Figure 1J,K). Through hematoxylin and eosin (H&E) staining, the thickening and fibrosis of the epidermis in the eccDNA treatment group were substantially reduced relative to the control group (Figure S6A, Supporting Information). The skin injury scores and irradiated areas in the eccDNA treatment group were notably lower compared to the control group (Figure 1L). The immunofluorescence assay showed that the eccDNA‐treated skin exhibited a 47.2% and 64% reduction in the levels of IL‐6 and TNF‐α expression, respectively (Figure 1M,N). Moreover, we conducted a comprehensive assessment of the impact of WS1 cells‐derived eccDNA treatment on the secretion levels of inflammatory factors from WS1 cells using an inflammation factor chip (Figure 1O and Figure S6E, Supporting Information), which demonstrated a reduction in the expression of various pro‐inflammatory factors (Figure S6B,C, Supporting Information). These findings suggest that eccDNA treatment significantly improves injury recovery and diminishes inflammation in radiation‐induced skin injury, highlighting its potential as a therapeutic strategy.

### 2.4 EccDNA Drives Increased Expression of VPS41

**Figure 2:**
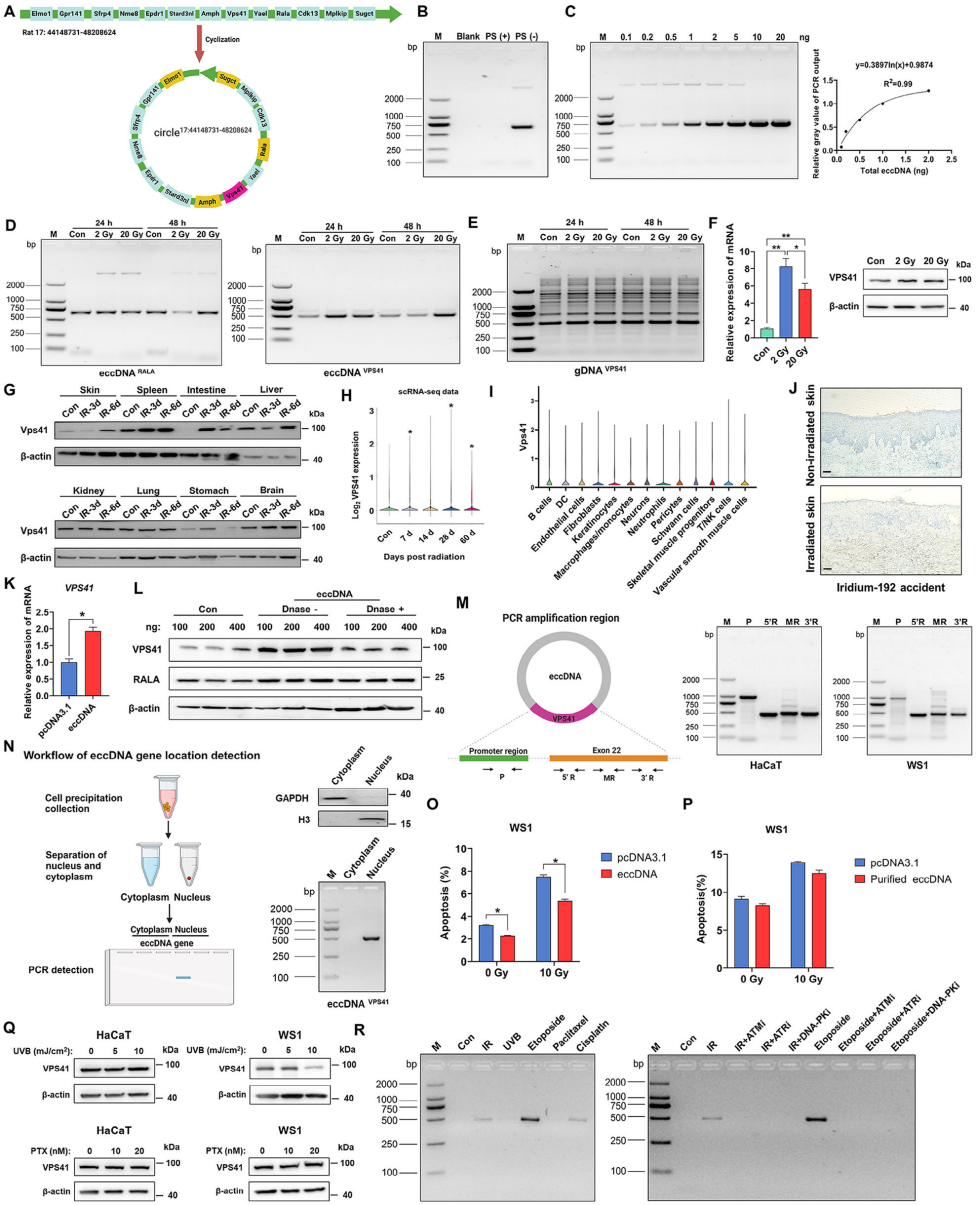
eccDNA drives increased VPS41 expression. A) Diagram of Circle^17:44148731‐48208624^ in rat skin. B) Validation of the PS enzyme for linear DNA removal. C) Standard curve for semiquantitative analysis of PCR by gel electrophoresis on the eccDNA gene. D) PCR analysis of RALA and VPS41 amplification in HaCaT cells post‐irradiation. E) Gel electrophoresis shows VPS41 copy number on gDNA remains unchanged after irradiation. F) Elevated mRNA and protein expression of VPS41 in HaCaT cells after irradiation. G) Vps41 protein expression in various tissues after 4 Gy (X‐ray) total body irradiation in rats. H) Vps41 expression (Log_2_ Fold Change) from scRNA‐Seq data of irradiated rat skin (*n* = 4 per group). I) Relative expression levels of Vps41 (Log_2_ Fold Change) in different cell types in scRNA‐Seq data of irradiated rat skin. J) Immunohistochemical analysis of VPS41 expression in a patient with clinical radiation‐induced skin injury (scale bar: 100 µm). K) Significant upregulation of VPS41 mRNA levels after transfection of HaCaT cells‐derived eccDNA into WS1 cells. L) Increased VPS41 expression after HaCaT cells‐derived eccDNA transfection into HEK‐293T cells. M) PCR analysis suggests intact gene expression elements for VPS41 on eccDNA. N) Nuclear‐cytoplasmic separation and semiquantitative analysis of PCR by gel electrophoresis reveal VPS41 gene localization on eccDNA. O) Apoptosis rate of WS1 cells transfected with total eccDNA after irradiation. P) Apoptosis rate of WS1 cells transfected with purified eccDNA after irradiation. Q) UVB and paclitaxel treatment effects on VPS41 expression in skin cells (HaCaT and WS1). R) Semiquantitative analysis of PCR by gel electrophoresis of DNA damage inducers and inhibitors on eccDNA^VPS41^ amplification in HaCaT cells. Treatments include IR (6 Gy), UVB (20 mJ/cm^2^), etoposide (2 µm), paclitaxel (20 nm), and cisplatin (2 µm). *P* values were calculated using different statistical methods based on data type: Mann–Whitney U test for two‐group comparisons and one‐way ANOVA followed by Bonferroni's post hoc test for multi‐group comparisons. Statistically significant differences are denoted as follows: ^*^ *p* < 0.05, ^**^ *p* < 0.01. Data are presented as mean ± SD (*n* = 3) unless otherwise specified.

To investigate whether the *VPS41* gene is similarly amplified on eccDNA post‐irradiation at the cellular level (**Figures** **2**A and S2A, Supporting Information), we initially tested *RALA* gene, which resides in the eccDNA of rat skin tissues and exhibited no obvious changes following irradiation, to validate the efficacy of the plasmid‐safe ATP‐dependent DNase (PS). The gel electrophoresis analysis of PCR products for *RALA* gene indicated that the PS effectively reduced gene amplification on the linear genome, highlighting PCR gel imaging as a reliable method of detecting eccDNA gene amplification (Figure 2B). In parallel, we constructed a standard curve based on the gel electrophoresis results derived from PCR products with varying concentrations of eccDNA template. A logarithmic function was generated by correlating the gray value detected via ImageJ software to the PCR product containing a 1 ng template, which implied that a loading range of 1–2 ng is optimal for eccDNA (Figure 2C). We selected 2 ng of eccDNA in rat skin tissue experiments to confirm whether *VPS41* and *RALA* were amplified on eccDNA. Our results indicated that VPS41 underwent significant amplification following irradiation (Figure 2D and Figure S6D, Supporting Information), whereas *RALA* did not exhibit similar changes (Figure 2D). In addition, VPS41 displayed no significant alterations in the genomic DNA following irradiation (Figure 2E).

Under identical irradiation conditions, the expression of VPS41 was upregulated at both the mRNA and protein levels (Figure 2F). This indicates that the enhancement of eccDNA^VPS41^ post‐irradiation is associated with elevated expression of *VPS41* gene. To comprehensively evaluate the impact of ionizing radiation on *VPS41* gene expression, we exposed rats to total body irradiation utilizing X‐rays, resulting in marked elevations of VPS41 expression in the irradiated dermis, spleen, small intestine, and gastric tissue (Figure 2G). Additionally, our previous scRNA‐Seq data^[^ ^33^ ^]^ from rat skin indicated an elevated expression of VPS41 following irradiation (Figure 2H). Notably, VPS41 expression levels remain relatively consistent across different cell types, suggesting that its radiation‐induced upregulation is a widespread phenomenon (Figure 2I). The immunohistochemistry staining of dermal tissue obtained from a patient exposed to a nuclear accident provided additional evidence for the heightened reaction of VPS41 to ionizing radiation (Figure 2J). To assess the direct impact of eccDNA on the expression of VPS41, we conducted HaCaT cells‐derived eccDNA transfection detection using the WS1 and HEK‐293T cell lines, showing a marked increase in both VPS41 mRNA and protein levels (Figure 2K,L). Moreover, the expression levels of VPS41 exhibited a notable decline following the hydrolysis of total eccDNA using DNase (Figure 2L). The aggregated results demonstrated that eccDNA promotes the expression of VPS41. Furthermore, we employed PCR to amplify the promoter of the longest exon 22 of *VPS41* present on eccDNA, encompassing the 5′ end, 3′ end, and central region. The subsequent gel electrophoresis results showed that the VPS41 located on eccDNA possesses a relatively intact set of gene expression elements (Figure 2M). Previous DNA‐FISH studies had successfully demonstrated the spatial localization of eccDNA genes; however, these probes were not widely applied to most non‐tumor genes. Consequently, we conducted semiquantitative analysis of PCR by gel electrophoresis following the segregation of nuclear and cytoplasmic fractions, indicating that eccDNA^VPS41^ is localized in the nucleus (Figure 2N). These findings demonstrate that eccDNA drives increased expression of VPS41, highlighting its role as a regulatory element in response to genotoxic stress.

### 2.5 Genotoxic Stress‐Induced Amplification of eccDNA^VPS41^ is a Consequence of DNA Breaks

Radiation can trigger the amplification of eccDNA^VPS41^, serving as an indicator of cellular internal demands. We found an additional elevation in total eccDNA rather than small eccDNA (purified by DNA beads) could enhance the cellular capacity to withstand apoptosis induced by radiation (Figure 2O,P). Due to the limitations of existing purification techniques, the size of purified eccDNA is mainly limited to parts smaller than 20 Kb,^[^ ^34^ ^]^ indicating that the eccDNA that exerts radiation protection is mainly composed of parts larger than 20 Kb, which is consistent with previous report that small eccDNA does not affect cell viability.^[^ ^28^ ^]^ Interestingly, we observed that other genotoxic treatments that induce cell death, such as ultraviolet B (UVB) radiation and paclitaxel, did not result in elevated VPS41 expression (Figure 2Q). To further elucidate the mechanism underlying the amplification of eccDNA^VPS41^, we performed a semiquantitative analysis of PCR by gel electrophoresis to measure the amplification of eccDNA^VPS41^ following various DNA damage inducers for genotoxic therapy. It was demonstrated that only etoposide, cisplatin, and X‐ray exposure could obviously trigger the amplification of eccDNA^VPS41^. A shared characteristic of these three modalities is their ability to directly induce DNA breakage (Figure 2R, Figures S6F and S7A–C, Supporting Information). Due to its capacity to selectively induce DNA breakage, etoposide showed the most potent ability to promote the amplification of eccDNA^VPS41^ (Figure 2R and Figure S7A–C, Supporting Information). In contrast to earlier studies^,[^ ^28^ ^]^ we employed various DNA damage inducers at relatively low concentrations, which did not trigger STING‐dependent innate immune responses (Figure S8A–H, Supporting Information).

### 2.6 VPS41 Confers Radioprotective Effects in Skin Cells

The selection of *VPS41* gene as an amplification target for radiation‐induced eccDNA in skin cells is intrinsically linked to its significant biological role. VPS41, a vacuolar sorting and lysosomal transport protein ^[^ ^35^ ^]^ mediates the conveyance of extracellular and intracellular signaling molecules, as well as other substances, from the plasma membrane to vacuoles or lysosomes for degradation.^[^ ^35^, ^36^ ^]^ Early research showed that VPS41 inhibits apoptosis pathways by blocking Caspase‐9, Caspase‐3 activation, and PARP cleavage, providing neuroprotection against toxins like 6‐OHDA and rotenone.^[^ ^35^ ^]^ Additionally, *VPS41* deficiency in β‐cells impairs insulin secretion, leading to diabetes.^[^ ^37^ ^]^

**Figure 3:**
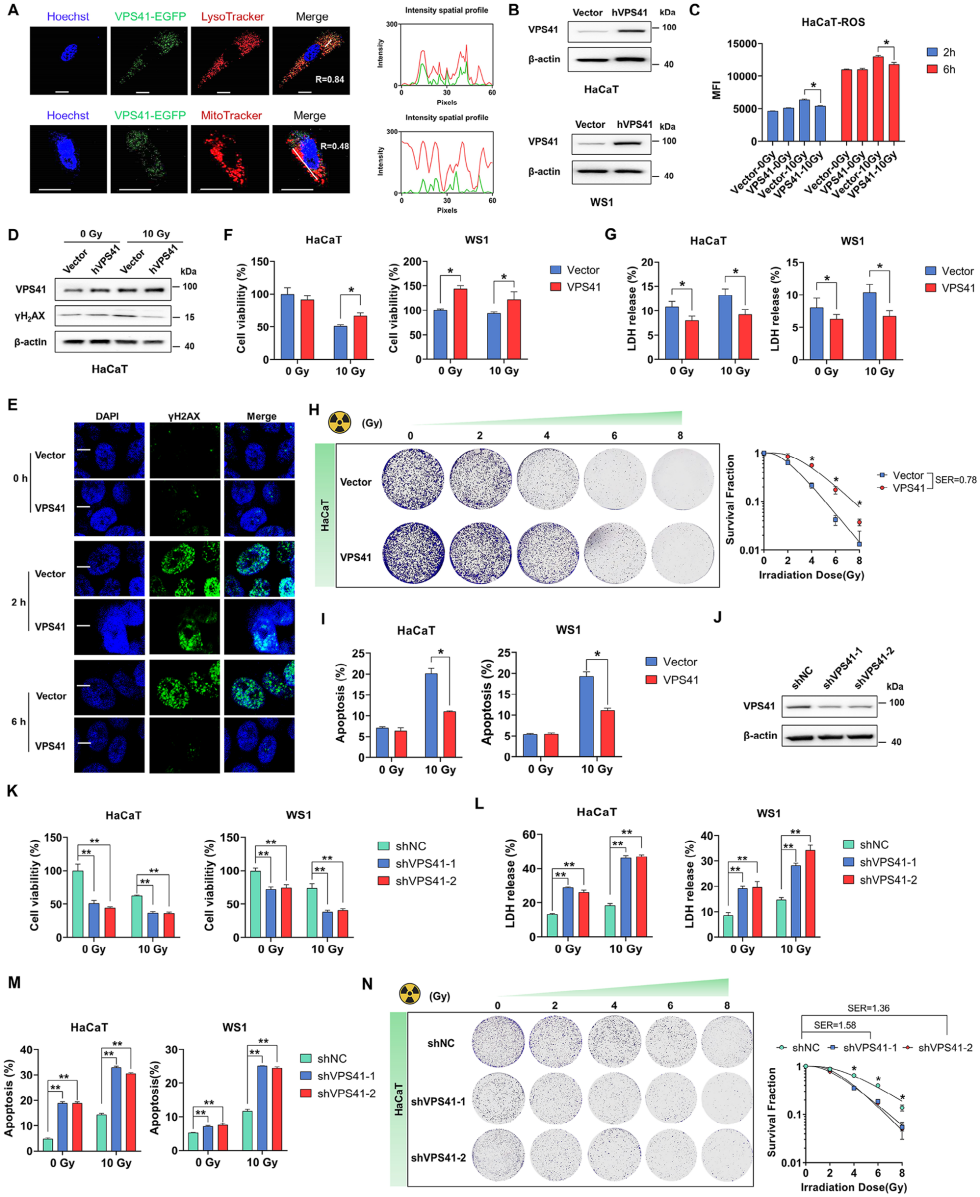
VPS41 upregulation confers radioprotective effects at the cellular level. A) VPS41 protein localization after EGFP‐VPS41 plasmid transfection in HaCaT and WS1 cells (Hoechst: blue, EGFP: green, LysoTracker: red; scale bar: 20 µm). B) Western blot showing VPS41 plasmid overexpression efficiency in HaCaT and WS1 cells. C) ROS levels in HaCaT cells post‐irradiation after VPS41 plasmid transfection. D,E) Effect of VPS41 plasmid transfection on γH2AX levels in HaCaT cells after irradiation (scale bar: 20 µm). F) Cell viability in HaCaT and WS1 cells post‐irradiation with VPS41 plasmid transfection. G) LDH release in HaCaT and WS1 cells following irradiation and VPS41 plasmid transfection. H) Colony formation rate in HaCaT cells post‐irradiation with VPS41 plasmid. I) Reduced apoptosis in HaCaT and WS1 cells post‐irradiation after VPS41 plasmid transfection. J) Western blot showing shVPS41 knockdown efficiency in HaCaT and WS1 cells. K) Decreased irradiated cell viability after shVPS41 infection in HaCaT and WS1 cells. L) Increased LDH release in irradiated HaCaT and WS1 cells post‐shVPS41 infection. M) Elevated apoptosis rate in irradiated HaCaT and WS1 cells after shVPS41 infection. N) Reduced colony formation in irradiated HaCaT cells after shVPS41 infection. *P* values were calculated using different statistical methods based on data type: Mann–Whitney U test for two‐group comparisons and one‐way ANOVA followed by Bonferroni's post hoc test for multi‐group comparisons. Statistically significant differences are denoted as follows: ^*^ *p* < 0.05, ^**^ *p* < 0.01. Data are presented as mean ± SD (*n* = 3) unless otherwise specified.

Initially, we validated the localization of VPS41 to lysosomes (**Figure** **3**A). To assess its potential radioprotective function, we examined the radiobiological effects of VPS41 upregulation in two skin cell types (HaCaT and WS1). Our findings demonstrated that overexpression of VPS41 (Figure 3B) led to a slight reduction in reactive oxygen species (ROS) levels (Figure 3C) and an obvious decrease in DNA damage, as assessed by Western blot analysis and γ‐H2AX staining (Figure 3D,E). And VPS41 overexpression increased cell viability by ≈1.3‐fold post‐irradiation (Figure 3F) and reduced lactate dehydrogenase (LDH) release by ≈30%, indicating improved membrane integrity (Figure 3G). This result was further supported by a substantial reduction in apoptotic cell death, with apoptosis rates falling by a mean 44% in the VPS41 overexpression group compared to controls (Figure 3I). The colony formation assay further corroborated these findings, revealing that VPS41 overexpression significantly increased the clonogenic survival of irradiated cells (Figure 3H). In contrast, knockdown of VPS41 expression through lentivirus‐mediated RNA interference (Figure 3J) resulted in a reversal of these protective effects. Specifically, cell viability was decreased by a mean of 41.6% in the VPS41 knockdown group (Figure 3K), LDH release increased by a mean of 1.8‐fold, as evidenced by a marked increase in the apoptosis rate, which increased by a mean of 1.2‐fold (Figure 3L,M), and the clonogenic survival decreased significantly (Figure 3N). Collectively, these findings indicate that the upregulation of VPS41 can enhance radioresistance in skin cells. Notably, we also discovered that the upregulation of VPS41 facilitated skin cell resistance to apoptosis induced by both paclitaxel and UVB radiation (Figure S6G,H, Supporting Information).

### 2.7 VPS41 Confers Radioprotective Effects In Vivo

**Figure 4:**
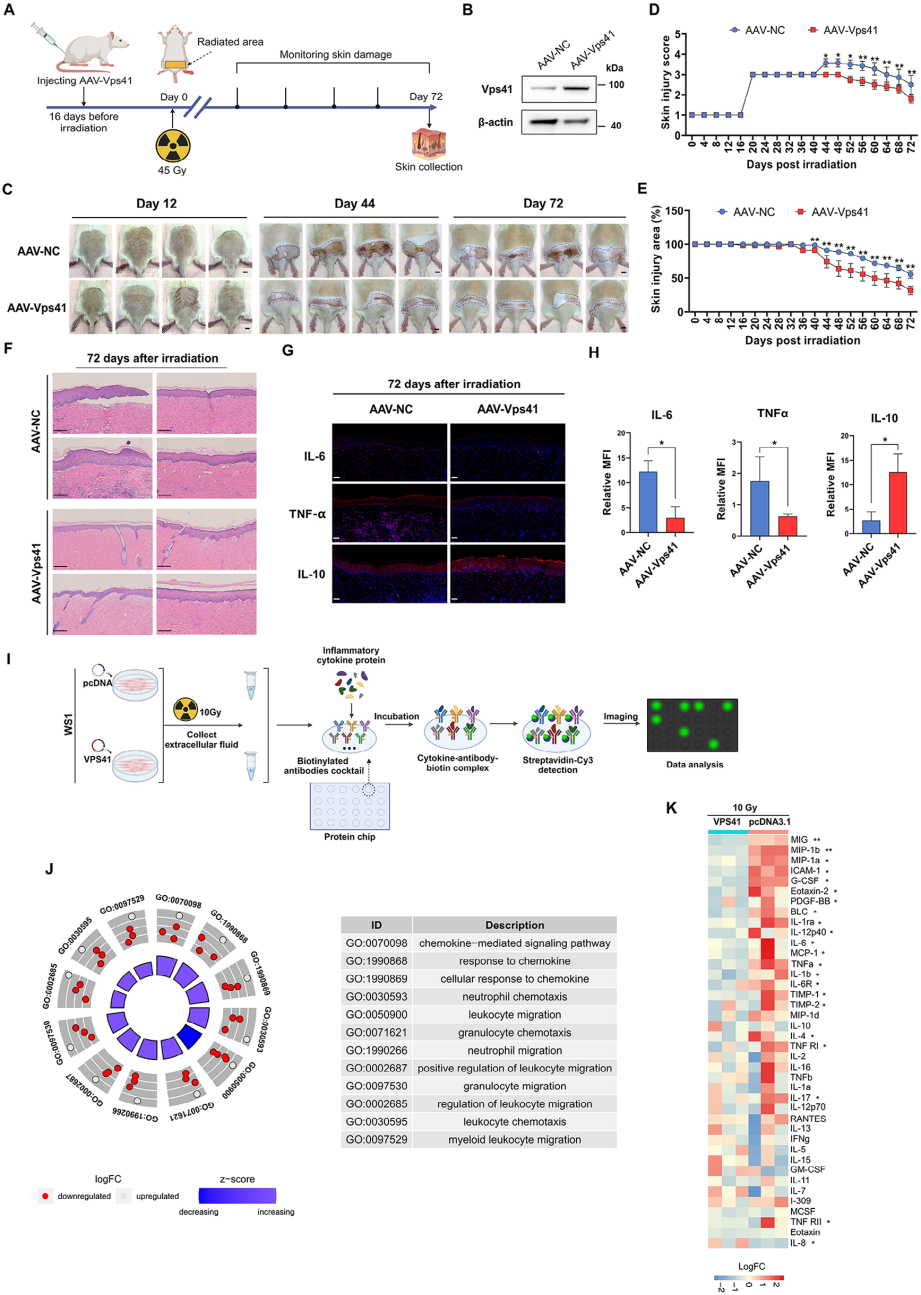
Therapeutic effects of AAV‐Vps41 on radiation‐induced skin injuries in rats. A) Flowchart for skin radiation injury in rats pre‐treated with AAV‐Vps41 for 16 days (*n* = 4 per group). B) Increased Vps41 protein expression in rat skin one month post AAV‐Vps41 infection. C) Photographs of skin radiation injury in AAV‐Vps41 pre‐treated rats on days 12, 44, and 72 post‐irradiation (scale bar: 1 cm). D) Radiation injury score statistics in AAV‐Vps41 pre‐treated rats. E) Analysis of the area of skin radiation injury in AAV‐Vps41‐treated rats. F) HE staining showing tissue resistance to ionizing radiation in AAV‐Vps41 pre‐treated rats (45 Gy for 72 days), scale bar 250 µm. G,H) Immunofluorescence detection and analysis of IL‐6, IL‐10, TNF‐α in irradiated skin of AAV‐Vps41 pre‐treated rats (scale bar: 100 µm). I) Schematic diagram of inflammatory factor chip detection in AAV‐Vps41 pre‐treated WS1 cells. J) GO classification of inflammatory factor chip results in AAV‐Vps41 pre‐treated WS1 cells. K) Heat Map of inflammatory factor chip results in AAV‐Vps41 pre‐treated WS1 cells. *P* values were calculated using different statistical methods based on data type: Mann–Whitney U test for two‐group comparisons, Fisher's exact test for GO enrichment analysis, and limma's empirical Bayes moderated t‐statistics for protein expression data. Statistically significant differences are denoted as follows: ^*^ *p* < 0.05, ^**^ *p* < 0.01. Data are presented as mean ± SD (*n* = 3) unless otherwise specified.

We used a rat model utilizing adeno‐associated virus Vps41 (AAV2/9‐Vps41, abbreviated as AAV‐Vps41 in the main text) to directly assess the radioprotective effects of VPS41, considering that Vps41 knockout in mice results in embryonic lethality (**Figure** **4**A,B). Our results indicated that the skin injury recovery level of the AAV‐Vps41 treatment group was significantly higher than that of the control group, demonstrating significant improvement in damage from day 44 post‐irradiation (Figure 4C). H&E staining results revealed that the epidermal thickening and fibrosis in the AAV‐Vps41 treatment group of rats were markedly reduced compared to the control group (Figure 4F). The AAV‐Vps41 treatment group exhibited reduced skin injury scores and injury areas compared to the control group (Figure 4D,E). Additionally, the immunofluorescence assay demonstrated that the eccDNA‐treated skin exhibited a 75.8% and 64% reduction in the levels of IL‐6 and TNF‐α expression, respectively, along with a 3.7‐fold increase in the expression of IL‐10 (Figure 4G,H). Moreover, we assessed the impact of AAV‐Vps41 treatment on the secretion levels of inflammatory factors in WS1 cells via an inflammation factor chip, which demonstrated a reduction in the expression of various pro‐inflammatory factors (Figure 4I–K).

Notably, VPS41 exhibited significantly different responses to genotoxic therapy in tumor cells compared to normal cells. Regardless of whether skin cells showed upregulation (specifically induced by ionizing radiation), downregulation, or no change in VPS41 expression following genotoxic treatment (such as X‐rays, UV exposure, or paclitaxel), upregulation of VPS41 consistently demonstrated a notable anti‐apoptotic effect (Figure 3I, Figure S8A,B, Supporting Information). In contrast, following genotoxic treatment of tumor cells (A375 and SiHa), there was minimal evidence of significant changes in VPS41 expression (Figure S9H–O, Supporting Information). These findings support the notion that increased VPS41 provides cells with a fundamental defense, offering widespread protective effects and potentially safety during genotoxic therapy.

### 2.8 VPS41 Mitigates Radiation‐Induced Apoptosis by Attenuating the KAI1 Via the Lysosomal Pathway

**Figure 5:**
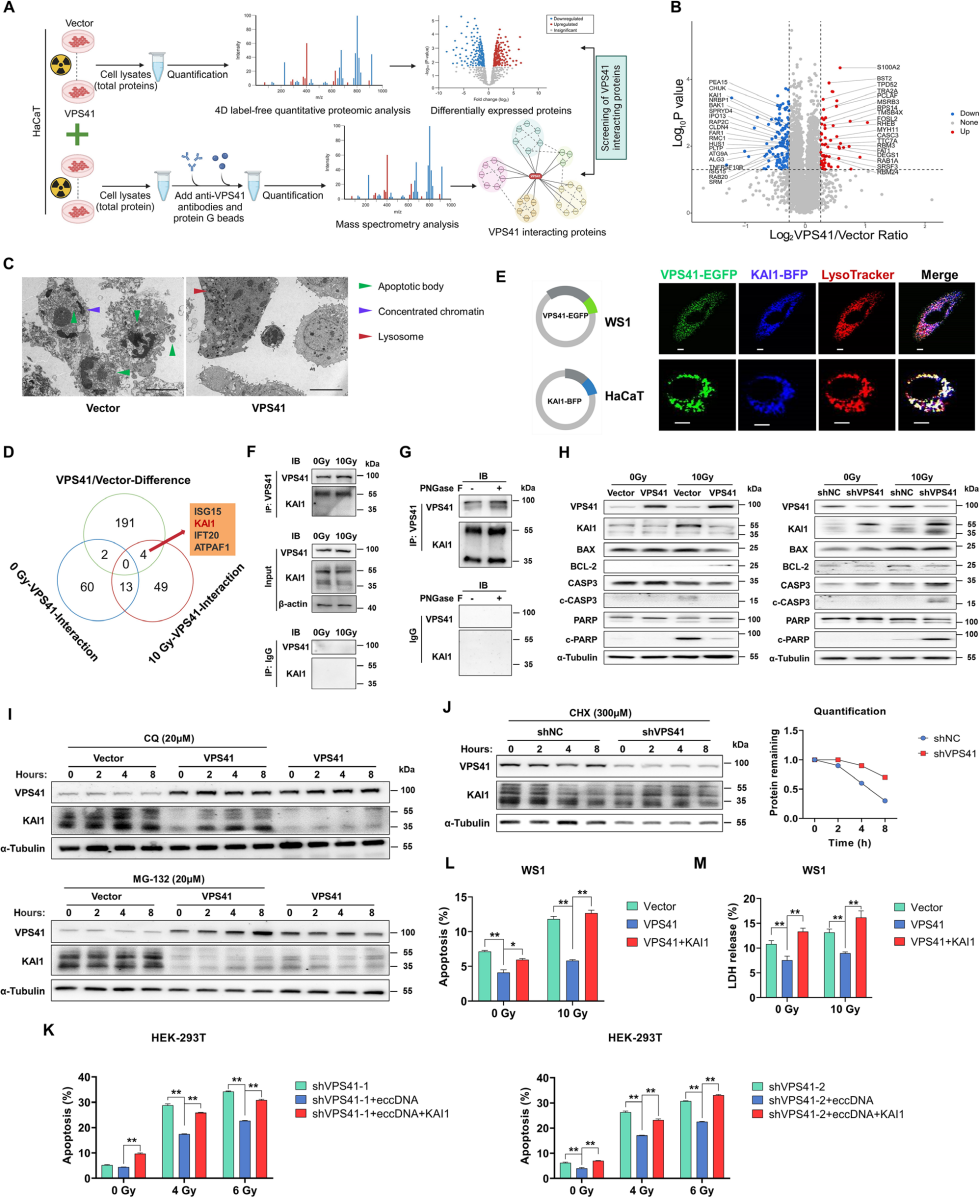
VPS41 negatively regulates KAI1 expression through the lysosomal pathway to confer resistance to apoptosis. A) Flowchart for screening VPS41 interaction proteins via differential protein analysis and mass spectrometry after VPS41 upregulation post‐irradiation. B) Volcano plot showing proteomic analysis (VPS41 vs Vector). C) Electron microscopy analysis reveals inhibited apoptosis progression in cells with upregulated VPS41 after irradiation (scale bar: 5 µm). D) Intersection of differential proteins identified four candidates: ISG15, KAI1, IFT20, and ATPAF1. E) Co‐localization of VPS41‐EGFP and KAI1‐BFP plasmids in WS1 and HaCaT cells assessed by confocal microscopy (scale bar: 20 µm). F) Immunoprecipitation confirms VPS41 binds KAI1. G) PNGase F treatment has minimal effect on VPS41‐KAI1 interaction. H) Western Blot shows upregulation of VPS41 decreases KAI1 expression, suppressing apoptosis, while VPS41 downregulation increases KAI1 expression and enhances apoptosis. I) VPS41 and KAI1 expression changes in HaCaT cells treated with CQ (20µm) or MG‐132 (20 µm) combined with X‐ray (10 Gy). J) Analysis of KAI1 decay rate after CHX (300 µm) treatment and X‐ray (10 Gy) in HaCaT cells. K) Effect of VPS41 knockdown and eccDNA transfection on apoptosis rates in irradiated cells with or without KAI1 overexpression. L) Apoptosis testing shows KAI1 reverses VPS41‐mediated radiation resistance. M) LDH measurement assesses the role of KAI1 in reversing VPS41‐mediated radiation resistance. *P* values were calculated using different statistical methods based on data type: unpaired two‐tailed t test for differential protein analysis and one‐way ANOVA followed by Bonferroni's post hoc test for multi‐group comparisons. Statistically significant differences are denoted as follows: ^*^ *p* < 0.05, ^**^ *p* < 0.01. Data are presented as mean ± SD (*n* = 3) unless otherwise specified. [Correction added on 28 April 2025, after first online publication: figure 5 is updated in this version].

In line with previous reports,^[^ ^35^, ^36^, ^37^ ^]^ VPS41 was predominantly located on the lysosomal membrane (Figure 2A). To explore the functional mechanism of VPS41 in radiation mitigation, we conducted a proteomic analysis of VPS41 upregulation in HaCaT cells exposed to a 10 Gy dose and examined the endogenous interacting proteome of VPS41 through mass spectrometry (**Figure** **5**A). The proteomic analysis revealed a total of 197 proteins with differential expression, comprising 138 proteins that were down‐regulated and 59 that were up‐regulated (Figure 5B). Notably, 38 of these differentially expressed proteins have direct or indirect associations with apoptosis (Figure 5B). We showed through electron microscopy that the overexpression of VPS41 markedly suppressed apoptosis (Figure 5C). To elucidate the molecular mechanism underlying the upregulation of VPS41, we identified the interacting proteins of endogenous VPS41 utilizing mass spectrometry (MS). The Venn diagram analysis of these proteins, alongside the differentially expressed proteins identified through proteomics, indicated that four candidate proteins were likely interacting partners of VPS41 (Figure 5D). Given the established functions of these proteins, we proposed KAI1 associated with apoptosis as a significant regulatory molecule for VPS41.

We further confirmed the direct interaction between VPS41 and KAI1 through co‐immunoprecipitation (Co‐IP) and co‐localization analysis (Figure 5E,F), revealing their co‐localization in lysosomes (Figure 5E). Additionally, we employed the glycosidase PNGaseF to explore whether the glycosylation on KAI1 influenced their interaction, and our findings indicated that its glycosylation did not appear to impact this process (Figure 5G). VPS41 exerted a negative regulatory effect on the protein expression of KAI1, consequently diminishing the levels of Caspase 3 and the cleavage of PARP (Figure 5H). These findings substantiate that VPS41 serves a protective role against radiation‐induced apoptosis through the down‐regulation of KAI1. Chloroquine (CQ) was shown to effectively counteract the negative regulation of KAI1 by VPS41, whereas the impact of MG132 was found to be less significant (Figure 5I). Furthermore, we employed cycloheximide (CHX) to assess the impact of VPS41 down‐regulation on the protein turnover rate of KAI1. The result indicated that VPS41 facilitated the degradation of KAI1 (Figure 5J). Additionally, the anti‐apoptotic function of VPS41 was reversed by KAI1 recovery assay (Figure 5L,M). Collectively, these findings suggest that VPS41 attenuates KAI1 levels via lysosomal degradation against apoptosis.

To investigate the regulatory role of eccDNA on KAI1 through the amplification of VPS41, we used HEK‐293T cells with VPS41 knockdown to avoid genomic DNA interference. Our findings showed that eccDNA transfection significantly mitigates radiation‐induced apoptosis, while KAI1 overexpression counteracted the protective effects of eccDNA, suggesting eccDNA regulated KAI1 expression through VPS41 amplification (Figure 5K). GEPIA2 database analysis indicated no correlation between VPS41 expression and various human malignancies, including skin cutaneous melanoma (SKCM) (Figure S10, Supporting Information). Additionally, VPS41 generally showed non‐significant or inverse association with patient survival across various cancers, including SKCM (Figure S11, Supporting Information).

### 2.9 The Interaction Between VPS41 and KAI1 is Critical for the Radioprotection of VPS41

**Figure 6:**
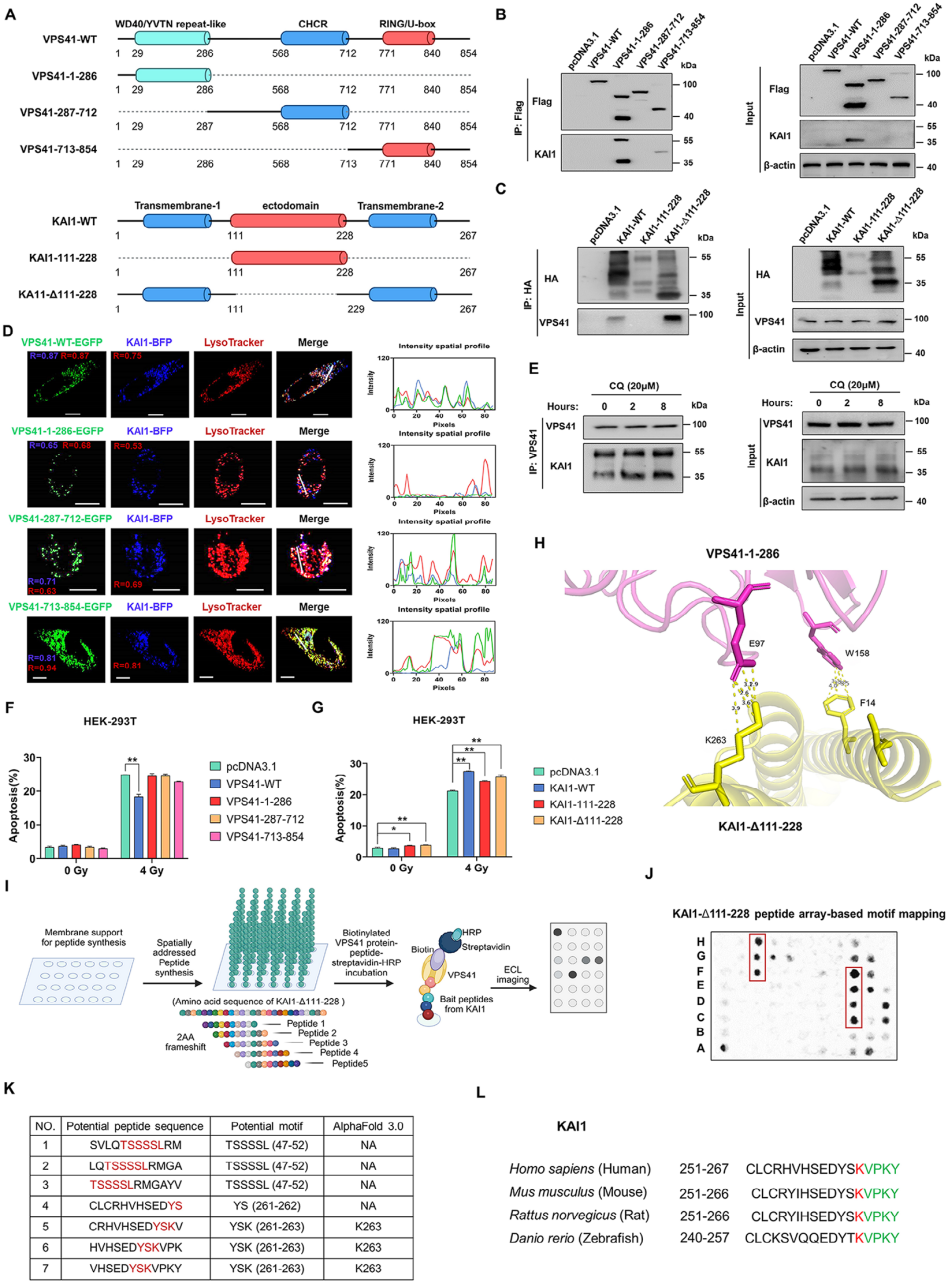
The interaction between VPS41 and KAI1 is critical for the radioprotection of VPS41. A) AlphaFold 3 prediction of structural domains for VPS41 and KAI1. B) IP experiments validate interaction domains between truncated VPS41 and KAI1 after transfection of various VPS41 truncation plasmids into HEK‐293T cells. C) IP experiments verify interaction domains between truncated KAI1 and VPS41 after transfection of KAI1 truncation plasmids into HEK‐293T cells. D) Co‐transfection of VPS41_WT‐EGFP and truncated variants with KAI1‐BFP in WS1 cells, followed by confocal microscopy to assess co‐localization (scale bar: 20 µm). E) The interaction between VPS41 and KAI1 remains unaffected by CQ treatment, which inhibits endosome and lysosome fusion. F) Apoptosis assays investigate the effects of truncated VPS41 on radiation‐induced apoptosis in HEK‐293T cells. G) Apoptosis assays assess the impact of different KAI1 truncation variants on radiation‐induced apoptosis in HEK‐293T cells. H) AlphaFold 3.0 predicts interaction sites of VPS41‐1‐286 and KAI1‐Δ111‐228. I) Schematic diagram of peptide array experiment. J) ECL imaging results of KAI‐Δ111‐228 peptide array. K) Peptide array and AlphaFold 3.0 analyze protein binding sites. L) Conservation of KAI1 binding peptide containing K263 among species. *P* values were calculated using one‐way ANOVA followed by Bonferroni's post hoc test for multi‐group comparisons. Statistically significant differences are denoted as follows: ^*^ *p* < 0.05, ^**^ *p* < 0.01. Data are presented as mean ± SD (*n* = 3) unless otherwise specified.

To characterize the interaction between VPS41 and KAI1 proteins, we analyzed various classic domains of VPS41 and KAI1 based on the Ensembl database (**Figure** **6**A). Co‐IP analysis revealed that the VPS41‐1‐286 sequence participated in the interaction with KAI1 (Figure 6B), whereas KAI1‐Δ111‐228 sequence was involved in its interaction with VPS41 (Figure 6C). Due to the low protein expression levels of KAI1 in HEK‐293T cells (Figure S12A,B, Supporting Information), no endogenous KAI1 expression was observed across the input groups (Figure 6C). Binding of VPS41‐1‐286 with KAI1 competitively inhibited the degradation of endogenous VPS41 by KAI1 (Figure 6B). Additionally, we constructed fusion proteins of these truncated forms with enhanced green fluorescent protein (EGFP) and blue fluorescent protein (BFP) to investigate their co‐localization (Figure 6D). Co‐staining of constructed proteins and lysosomes demonstrated that the VPS41‐1‐286 sequence was responsible for binding with KAI1, while the VPS41‐713‐854 sequence was sufficient to induce lysosomal distribution of EGFP, suggesting it contains lysosomal sorting sequences (Figure 6D). Based on the role of CQ as an inhibitor of fusion between the plasma membrane and lysosomal membrane, the Co‐IP experiments conducted following CQ treatment indicated that the interaction between VPS41 and KAI1 is independent of membrane fusion (Figure 6E). Furthermore, the different domains of VPS41 and KAI1 in radiation‐induced apoptosis assays demonstrated that only the full‐length VPS41 exhibited radioprotection (Figure 6F and Figure S12C, Supporting Information), while both the extracellular and intracellular regions of KAI1 exacerbated radiation‐induced apoptosis (Figure 6G and Figure S12D, Supporting Information). The results indicate that VPS41 interacts with the intracellular domain of KAI1 through its N‐terminus and promotes lysosomal pathways via the C‐terminus.

To clarify the interaction details between VPS41 and KAI1, we utilized AlphaFold 3.0 to predict the binding sites of the two proteins (Figure 6H). Subsequently, considering the challenge of obtaining full‐length protein KAI1 as it is a poorly studied transmembrane protein, we identified the binding sites using the peptide array of KAI1‐Δ111‐228 (Figure 6I,J, Figure S13A,B, Supporting Information). Both the peptide array results and AlphaFold predictions indicated that the K263 residue serves as a reliable binding site (Figure 6J,K). Additionally, the KVPKY motif containing the K263 residue was conserved across species (Figure 6L). These findings provide compelling evidence for the critical role of the KAI1 K263 residue in the VPS41‐KAI1 interaction and offer insights into its evolutionary conservation.

## 3 Discussion

The role of extrachromosomal circular DNA (eccDNA) in disease diagnosis and progression has gained increasing attention, especially in oncogene amplification and cancer therapy resistance.^[^ ^24^, ^25^, ^38^, ^39^, ^40^, ^41^ ^]^ EccDNA can independently amplify key oncogenes like EGFR and Myc, driving tumor heterogeneity and aggressiveness, particularly under genotoxic stress.^[^ ^39^, ^41^ ^]^ This amplification, often associated with mechanisms such as chromothripsis, non‐coding regulatory elements, and coordinated inheritance, enables the persistent transcription of oncogenes, enhancing tumor resistance to therapies like chemotherapy and radiotherapy.^[^ ^42^, ^43^ ^]^ For example, oncogene amplification in eccDNA has been linked to resistance to methotrexate via DHFR amplification.^[^ ^44^, ^45^ ^]^ Importantly, high eccDNA levels, particularly those linked to oncogenes, correlate with poor prognosis, suggesting that eccDNA may serve as a useful biomarker and a potential therapeutic target in cancer.^[^ ^46^, ^47^ ^]^ However, the physiological roles of eccDNA in normal tissues, particularly under genotoxic stress, remain largely unexplored. In contrast to the high copy number of oncogene‐associated eccDNA in tumors, normal tissues, such as skin, exhibit low eccDNA levels under baseline conditions, as observed through Circle‐Seq analysis. In our study, we identified 150 potential dysregulated eccDNA genes in rat skin. Gene Ontology (GO) enrichment analysis revealed that these genes are primarily involved in regulating proteolytic enzymes, a notable distinction from tumor‐derived eccDNA, which typically selects for oncogenes that promote cell proliferation.^[^ ^48^, ^49^ ^]^ Interestingly, our study demonstrates that eccDNA protected normal cells from genotoxic stress by normalizing cytokine levels, which is consistent with reports suggesting that eccDNA contributes to cellular resilience.^[^ ^48^, ^49^ ^]^ Our findings highlight a previously underappreciated aspect of eccDNA biology, suggesting that it may play a critical role in maintaining cellular homeostasis under genotoxic stress and contributing to tissue resilience.

We identified a significant increase in eccDNA abundance and composition following ionizing radiation exposure, with a notable amplification of the eccDNA region containing the VPS41 gene. The identification of VPS41 as the sole amplified gene on eccDNA in irradiated tissues suggests a targeted regulatory mechanism. Such specificity might reflect the biological necessity of maintaining tissue integrity and cellular homeostasis under genotoxic stress. Significantly, we observed that in rat skin, only 5 of 13 genes in the identified eccDNA region retained intact coding sequences, suggesting that eccDNA may undergo structural alterations, such as deletions, even though the circular DNA junctions are confirmed to be present. These deletions are likely a result of chromatin crosslinking, a known consequence of genotoxic stress.^[^ ^50^, ^51^ ^]^

Our most noteworthy finding is the identification of VPS41 as a novel radioprotective gene within the eccDNA landscape. VPS41 is a conserved protein involved in vacuolar sorting and lysosomal trafficking, and its role in transporting signaling molecules for degradation is well documented.^[^ ^52^, ^53^, ^54^ ^]^ We confirmed the amplification of VPS41 in radiation‐induced eccDNA and identified a novel protective function in dermal cells exposed to genotoxic stress. VPS41 upregulation in normal skin cells following ionizing radiation contrasts with its stable expression in tumor cells, suggesting a differential response to genotoxic stress in normal versus cancerous tissues. Importantly, exogenous VPS41 appears to confer anti‐apoptotic protection, suggesting its potential for protecting normal tissues from radiotherapy‐induced damage. This differential regulation of VPS41 in response to genotoxic stress between normal and cancerous tissues may offer a novel therapeutic avenue for minimizing collateral damage to healthy tissues during radiotherapy.

**Figure 7:**
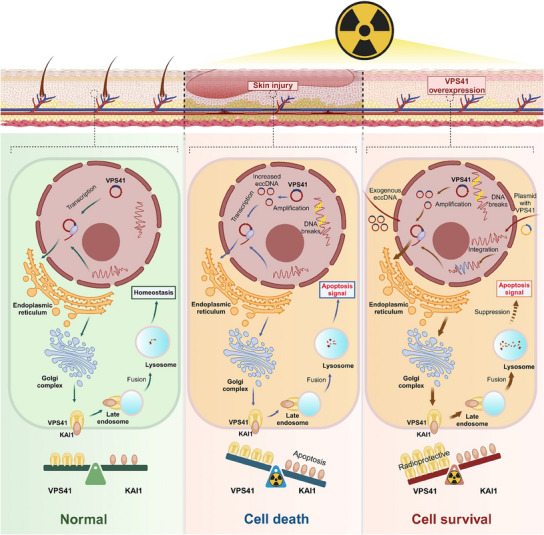
Schematic representation of the eccDNA‐VPS41‐KAI1 axis under ionizing radiation exposure. Under normal conditions, VPS41 and KAI1 expression in skin cells is tightly regulated. Upon ionizing radiation exposure, VPS41 is amplified on eccDNA, leading to a slight increase in its mRNA and protein levels, but insufficient to prevent radiation‐induced apoptosis. The introduction of exogenous eccDNA or VPS41 enhances VPS41 expression, promoting its interaction with KAI1 on the cell membrane. This interaction triggers lysosomal degradation of KAI1, suppressing apoptotic signaling and improving cell viability and resistance to radiation‐induced injury.

A pivotal discovery of this study is the mechanistic insight into how VPS41 exerts its radioprotective effects through its interaction with KAI1, a known metastasis suppressor protein.^[^ ^55^, ^56^, ^57^ ^]^ VPS41 specifically promotes the lysosomal degradation of KAI1, thereby mitigating the pro‐apoptotic and pro‐inflammatory pathways activated during radiation‐induced cellular stress. This interaction was validated by peptide array assays and structural modeling, which identified the binding domains critical for the VPS41‐KAI1 interaction. The degradation of KAI1 appears to fine‐tune cellular homeostasis by reducing stress‐related signaling cascades, ultimately preserving tissue integrity under genotoxic stress. From a biomedical perspective, this finding has several implications. First, it highlights the dual functionality of VPS41 in normal tissue protection and cellular stress adaptation. While KAI1 has been predominantly studied for its tumor‐suppressive properties ^[^ ^55^, ^56^, ^57^ ^]^ its role in normal tissue homeostasis and stress responses remains underexplored. Our study demonstrates that the controlled degradation of KAI1 by VPS41 is essential for maintaining cellular viability and reducing radiation‐induced damage, suggesting that KAI1 may act as a “stress amplifier” under certain conditions. Second, this interaction provides a novel therapeutic axis for radioprotection. Pharmacologically targeting VPS41‐KAI1 interactions could allow for selective modulation of stress response pathways, enhancing the therapeutic index of genotoxic cancer therapies. Finally, given the divergent roles of KAI1 in tumor suppression and normal tissue protection, this mechanism underscores the importance of tissue‐specific regulatory networks and the need for precision medicine approaches to exploit these pathways effectively. By uncovering the VPS41‐KAI1 regulatory axis (**Figure** **7**), this study not only elucidates a key protective mechanism against radiation‐induced injury but also opens new avenues for therapeutic intervention aimed at reducing the collateral damage of radiotherapy.

In conclusion, this study identifies VPS41 as a key eccDNA gene that amplifies in response to genotoxic stress as part of a cellular defense mechanism. Our findings reveal a novel radioprotective mechanism in which VPS41 promotes the lysosomal degradation of KAI1, preventing its accumulation and thereby alleviating radiation‐induced damage (Figure 7). These findings open new avenues for exploring eccDNA‐based strategies to alleviate tissue damage in genotoxic therapies and enhance therapeutic efficacy through targeted modulation of cellular defense pathways.

## 4 Experimental Section

### Reagents

Paclitaxel, etoposide, cisplatin, cycloheximide (CHX), chloroquine (CQ), MG132, AZD6738, and AZD‐7648 were sourced from MCE (Monmouth Junction, NJ), while KU‐55933 was acquired from Sigma–Aldrich (St. Louis, MI).

### Sample Preparation for Circle‐Seq

Male Sprague‐Dawley (SD) rats, 8 weeks old, were sourced from Chengdu Dossy Experimental Animals Co., Ltd (Chengdu, China). The rats were housed in a controlled environment (21 °C ± 1 °C, 55 ± 10% humidity, 12‐h light/dark cycle). Anesthesia was induced by intraperitoneal injection of 350 mg k^−1^g 10% chloral hydrate. Hair was shaved from the dorsal and gluteal regions, and the rats were secured with adhesive tape on a plastic platform to minimize movement during radiation exposure. A 3‐cm‐thick lead shield was used to define the radiation field (3 × 4 cm). Irradiation was administered at 1000 cGy min^−1^ using a 6 MeV electron beam (Clinac 2100EX; Varian Medical Systems, Palo Alto, CA) or 1.7 Gy min^−1^ with an X‐ray linear accelerator (KUBTEC XCELL 320, Milford, CT), as reported previously.^[^ ^58^, ^59^ ^]^ For Circle‐Seq detection, rats received fractional electron beam radiation to the dorsal and gluteal skin with a one‐week interval between sessions. Following irradiation, the rats were euthanized, and tissues from the irradiated areas were collected for analysis. The study was approved by the Animal Experimentation Ethics Committee at Sichuan University (Chengdu, China).

### Cell Culture and Irradiation

The HaCaT (human keratinocyte) cell line was obtained from the German Cancer Research Center (Heidelberg, Germany) as previously reported^,[^ ^60^ ^]^ and the WS1 (human skin fibroblast) cell line was purchased from ATCC.^[^ ^58^ ^]^ HEK‐293T, A375, and SiHa cell lines were purchased from Cellcook (Guangzhou, China).^[^ ^61^ ^]^ All cell lines were tested and verified mycoplasma‐free. Cells were cultured in DMEM supplemented with 10% FBS (Gibco, Carlsbad, CA) and 100 U mL^−1^ penicillin‐streptomycin, in a humidified incubator at 37 °C and 5% CO^2^. Cells were irradiated with varying doses (2, 4, 5, 6, 10, or 20 Gy) using an X‐ray linear accelerator (KUBTEC XCELL 320, Milford, CT) at a dose rate of 1.7 Gy min^−1^, as previously reported.^[^ ^59^ ^]^

### Crude eccDNA Isolation

Each rat skin sample was divided into small pieces using a chopper and weighed at 20mg. In brief, rat skin samples or human skin cells (HaCaT and WS1, 1×10^7^ per sample) were resuspended in 0.6 mL L1 solution (Plasmid Mini AX; A&A Biotechnology) and supplemented with 15 µL Proteinase K (Vazyme, Nanjing, China), followed by overnight incubation at 50 °C with agitation (200 rpm). Samples underwent alkaline treatment to separate chromosomal DNA, lipids, and proteins from eccDNAs, followed by column chromatography on an ion exchange membrane (Plasmid Mini AX; A&A Biotechnology). DNA was precipitated by incubating at −20 °C for 45 min, then centrifuging at 9788 × g for 30 min at 4 °C. Precipitated DNA was dissolved in 55 µL water to achieve concentrations of 1–40 ng µL^−1^. Crude eccDNA concentration was measured using a Qubit dsDNA HS Assay Kit (Invitrogen, Carlsbad, CA). For localization detection of eccDNA gene, crude eccDNA samples were subjected to nuclear isolation using the Minute Detergent‐Free Nuclei Isolation Kit (NI‐024, Invent, MI) prior to extraction.

### Removal of Linear DNA and eccDNA Purification for Circle‐Seq

To remove the linear portions of crude eccDNA of rat skin samples, 25 µL eccDNA was treated with 20 units of Plasmid‐Safe ATP‐dependent DNase (Invitrogen, Carlsbad, CA) at 37 °C for 24 h, as reported previously.^[^ ^28^ ^]^ The Plasmid‐Safe ATP‐dependent DNase was then inactivated by incubating samples at 70 °C for 30 min. The digestion products were cleaned up from the reaction mixes using 1.8 × AMPure XP beads (Beckman Coulter, Brea, CA) and eluted in 30 µL RNase‐free water and quantified by Qubit dsDNA HS Assay Kit (Thermo Fisher, Waltham, MA) for further Circle‐Seq detection.

### Screening and Validation of eccDNA from Circle‐Seq Data

Stringent selection criteria was applied to identify 40 eccDNAs exhibiting both split and discordant reads (Tables S2 and S3, Supporting Information). Each selected eccDNA met a minimum threshold of 10 for either split or discordant reads, with a total read count (split + discordant reads) of at least 20. These eccDNAs were subsequently chosen for experimental validation. EccDNA validation was performed by outward PCR and Sanger sequencing, with primers listed in Table S4 (Supporting Information). Each 50 µL PCR reaction contained exonuclease‐treated template (4 µL), 200 nm primer, dNTP, buffer, and 2× Taq Plus Master Mix. PCR was conducted for 30 cycles using a PCR cycler (Heal Force GM‐05, Shanghai, China) under the following conditions: 95 °C for 5 min, 30 cycles of 95 °C for 15 s, 60 °C for 30 s, 72 °C for 1 min, followed by final elongation at 72 °C for 5 min and storage at 4 °C. PCR products were visualized on 1.5% agarose gels with a Luminescent Image Analyzer (LAS‐4000 Mini, Tokyo, Japan). Specific positive bands were purified, cloned using TOPO‐TA cloning (Zero TOPO‐TA Cloning Kit, Yeasen, Shanghai, China), and sent for Sanger sequencing (Youkang Biotechnology Zhejiang Co., Ltd, Zhejiang, China). Sanger sequencing confirmed the junction site of all selected eccDNAs. The 278 bp region spanning the junction site of ecc34 was used as a quality control, as it can be detected in PCR products when crude eccDNA (CE) serves as the template, corresponding to its linear genomic counterpart. However, its detection was significantly reduced in PCR products when exonuclease‐treated eccDNA (EE) was used as the template across all samples. The PCR primers for the ecc34 junction region were as follows: Forward: 5′‐GGGGCCCACACAGGGTGGGCACCTG‐3′; Reverse: 5′‐AGTGCTCCACCCTGTGTGA

GTCCTT‐3′. For the corresponding linear gene region of ecc34 (chr20:46819475–46819752), a 278 bp segment was selected, and the PCR primers are: Forward: 5′‐CAATAACCGCAACGTTTTCCAGTGT‐3′; Reverse: 5′‐GCTAGAATAAACACACCTGTAAGTCTTTGAC‐3′.

### Semiquantitative Analysis of PCR by Gel Electrophoresis on eccDNA Genes

The eccDNA of different template amounts was detected by agarose gel electrophoresis, and the gray value was calculated using Image J software to determine the standard curve for relative PCR yield. A total of 2 ng of eccDNA template was used for PCR‐based detection of eccDNA genes. The amplification protocol followed the same conditions as those used for eccDNA validation. PCR primers for eccDNA genes are listed in Tables S5 and S6 (Supporting Information). PCR products were resolved by 1.5% agarose gel electrophoresis and visualized with GelBlue (Yeasen, Shanghai, China) staining.

### Statistical Analysis

Data were presented as mean ± SD (*n* = 3) unless otherwise specified. Statistical analysis was conducted using GraphPad Prism 9.5 software (GraphPad Software, Inc., La Jolla, CA), as detailed in the results section. *P* values were determined using appropriate statistical methods according to the analysis type: Mann–Whitney U test (two‐tailed) for two‐group comparisons; one‐way ANOVA followed by Bonferroni's post hoc test for multi‐group comparisons; Fisher's exact test for GO enrichment analysis (one‐tailed); limma's empirical Bayes moderated t‐statistics (two‐tailed) for differential protein analysis of protein chip data; and unpaired two‐tailed t test for differential protein analysis of proteome data. Statistically significant differences were denoted as follows: ^*^ *p* < 0.05, ^**^ *p* < 0.01.

### Supplemental Methods

An extended methods section is available in Supporting Information.

## Conflict of Interest

The authors declare no conflict of interest.

## Author contributions

S.Z. did the conceptualization. B.S., P.Y., H.Q., and Y.Z. did the methodology. B.S., J.H., and B.S. did an investigation. F.D., Y.T., and Q.L. did visualization. S.Z. and D.Y. did funding acquisition. C.S. and L.T. did project administration. S.Z. and W.T. did the supervision. S.Z. and B.S. did write‐original draft. H.B. and D.Y. did writing‐review & editing.

## Supporting information



Supporting Information

## Data Availability

The data that support the findings of this study are openly available in GEO at https://www.ncbi.nlm.nih.gov/geo/query/acc.cgi?acc=GSE218553, reference number 218553.
