# Supplementary material for: EccDNA‐Driven VPS41 Amplification Alleviates Genotoxic Stress via Lysosomal KAI1 Degradation
Source: Adv Sci (Weinh). 2025 Apr 24;12(25):2501934. doi: 10.1002/advs.202501934 (PMC12224964; doi:10.1002/advs.202501934)
Supplement: Supplementary file 1 — Supporting Information [file ADVS-12-2501934-s001.docx]

**Supporting Information for**

**EccDNA-driven VPS41 amplification alleviates genotoxic stress via lysosomal KAI1 degradation**

Bin Shi, Ping Yang, Huaijin Qiao, Jinchen He, Bin Song, Hao Bai, Fengdi Jiang, Yining Zhang, Qian Li, Tao Yan, Wenlin Tu, Daojiang Yu, Shuyu Zhang

Corresponding author: Shuyu Zhang.

E-mail：[zhang.shuyu@hotmail.com](mailto:zhang.shuyu@hotmail.com)

**This file includes:**

Supplemental methods

Supplemental results

References

**Supplemental methods**

**Library preparation and eccDNA sequencing for Circle-Seq**

According to the manufacturer's instructions, the Illumina sequencing library of eccDNA was prepared using the Nextera XT DNA sample preparation Kit and labeling method based on Tn5 transposon. Subsequently, 50 ng of eccDNA was used for library preparation on the Illumina sequencing platform, performed by Genedenovo Biotechnology Co., Ltd. (Guangzhou, China). The length distribution and quality of each library were assessed using an Agilent 2100 (Agilent, Santa Clara, CA). Finally, the DNA libraries were sequenced using the NovaSeq 6000 Sequencer (Illumina, San Diego, CA) with a paired-end 150 bp (PE150) strategy.

**Filtering of Circle-Seq data**

To ensure data quality, raw reads were filtered using fastp [1], removing low-quality data and generating clean reads. The filtering steps included: 1) removing adapter-containing reads; 2) removing reads with >10% N content; 3) removing reads composed entirely of A bases; 4) removing low-quality reads (where Q ≤ 20 for >50% of the read).

**Annotation and basic analysis of Circle-Seq data**

Sequencing reads were aligned to the human reference genome (hg38, UCSC) using BWA-MEM. Two BAM files were sorted by read name and coordinates to extract circular reads. Genomic annotation data were downloaded from the UCSC table browser ([https://genome.ucsc.edu/cgibin /hgTables/](https://genome.ucsc.edu/cgibin%20/hgTables/)). EccDNAs were mapped to seven genomic element classes (exon, intron, 2 Kb upstream and downstream of genes, intergenic). Bedtools 2.29.2 was used to extract 10 bp upstream and downstream sequences of each eccDNA junction site, and HomerTools calculated the mean per base mononucleotide frequencies. Motif signatures at eccDNA junctions were visualized using the R package ggseqlogo-0.1.

**Identification and quantification of eccDNA from Circle-Seq data**

We employed Circle-Map software to detect eccDNA from Circle-Seq data [2]. The algorithm first identifies circular DNA junctions by detecting discordant read pairs spanning the circular DNA boundary. This initial mapping is refined using soft-clipped reads (split reads), which provide higher resolution for junction localization. The integration of these two read types enhances the accuracy of eccDNA identification. For quantitative analysis, we used eccDNA counts (split reads + discordant reads) to quantify and compare the levels of eccDNA.

**Isolation and hydrolysis of eccDNA for transfection**

For the transfection experiments with eccDNA, all tissues (20mg per sample) and cells (1 × 10⁷ per sample) were subjected to eccDNA isolation following the standard protocol for crude eccDNA extraction as described in Method section. The enzymatic digestion of linear genomic DNA from crude eccDNA was conducted using the same pre-treatment protocol as for Circle-Seq. The subsequent steps were performed according to established procedures reported in the literature [3]. Specifically, crude eccDNA was digested with PacI (NEB, Ipswich, MA) for 4 hours to hydrolyze mitochondrial DNA. For eccDNA transfection into HEK-293T cells, total eccDNA was hydrolyzed by treating the samples with DNase I (Vazyme, Nanjing, China) for 1 hour. Crude eccDNA treated with PacI was then purified using phenol: chloroform: isoamyl alcohol (PCI) (25:24:1) in phase-lock gel tubes, followed by ethanol precipitation with carrier glycogen (Roche) and 1/10 volume of 3 M sodium acetate (pH 5.5). The precipitated crude eccDNA was resuspended in Solution A (One-Step Max Plasmid DNAout, TIANDZ, Beijing, China), where eccDNA selectively bound to magnetic silica beads and was subsequently eluted using 0.1× elution buffer (1 mM Tris-HCl, pH 8.0). EccDNA concentration was measured using the Qubit dsDNA HS Assay Kit (Thermo Fisher, Waltham, MA).

For transfection experiments in WS1 and HEK-293T cells, eccDNA isolated from HaCaT or WS1 cells exposed to 10 Gy irradiation for 24 hours was used. For transfection in rat skin tissues, eccDNA was derived from rat skin exposed to 5 x 4 Gy irradiation.

**RNA isolation and real-time PCR**

Total RNA was isolated from human cells using TRIzol (Invitrogen, Carlsbad, CA) in accordance with the guidelines provided by the manufacturer. To quantify the transcripts of interest genes, real-time PCR was performed using a SYBR Green Premix Ex Taq (Takara, Shiga, Japan) on Light Cycler480 (Roche, Basel, Switzerland). Primers were listed as follows: VPS41 (Forward: 5′-CAGTGCTAAGAACCGTGGAC-3′; Reverse: 5′-GCAAGAATCGTAGCCAGCA-3′); KAI1 (primer 1) (Forward: 5’-ATCTTCTTTATCCTGG GCGCAGTGA-3′; Reverse: 5’-GTTGTC TGTCCAGTTGTAGAAGCTG-3′); KAI1 (primer 2) (Forward: CTTCTACTTCAACATGGGCAAGCTG-3′; Reverse: 5’-CTGTATCTTCGGAAT GGACGTGCC-3′); GAPDH (Forward: 5′-CAACGGATTTGGTCGTATT-3′; Reverse: 5′-CACAGTCTTCTGGGTGGC-3′). Relative quantitation analysis of gene expression data was conducted according to the 2^−ΔΔCt^ method.

**Plasmids construction and transfection**

Plasmid construction followed a previously established protocol [4]. The pcDNA3.1-Flag-VPS41 (NM_014396.4) expression vector was generated by inserting synthesized cDNA encoding Flag tag and VPS41 into the pcDNA3.1 vector between EcoRI/XhoI sites (PPL, Nanjing, China). The pcDNA3.1-HA-KAI1-BFP (NM_002231.4) vector was constructed by inserting cDNA encoding an N-terminal Flag tag, C-terminal BFP tag, and KAI1 into the pcDNA3.1 vector, using EcoRI/XhoI (PPL, Nanjing, China). Truncated VPS41 and KAI1 plasmids were similarly generated by inserting cDNA encoding Flag-tagged VPS41 (WT, 1-286 AA, 286-712 AA, 713-854 AA) and HA-tagged KAI1 (WT, 111-228 AA, Δ111-228 AA) into pcDNA3.1 using EcoRI/XhoI (PPL, Nanjing, China). All plasmids were sequenced in both forward and reverse orientations to verify the correct sequence and absence of unintended mutations.

Plasmids and eccDNA were transfected into human cells using Lipofectamine 3000 (Thermo Fisher Scientific, Waltham, MA) per standard protocols. EccDNA was transfected into rat subcutaneous tissue using Entranster™-in vivo (Engreen Biosystem Co, Ltd., Beijing, China).

**Sequence and sources of lentiviruses and adeno-associated virus**

For in animal study, Vps41 (NM_001399318.1) overexpression adenovirus**-**associated (AAV2/9-Vps41, abbreviated as AAV-Vps41 in the main text) was obtained from HanBIO Tech (Shanghai, China). Sequences on lentiviruses related with this manuscript listed as follows. Infections were adopted according to the standard procedures.

**
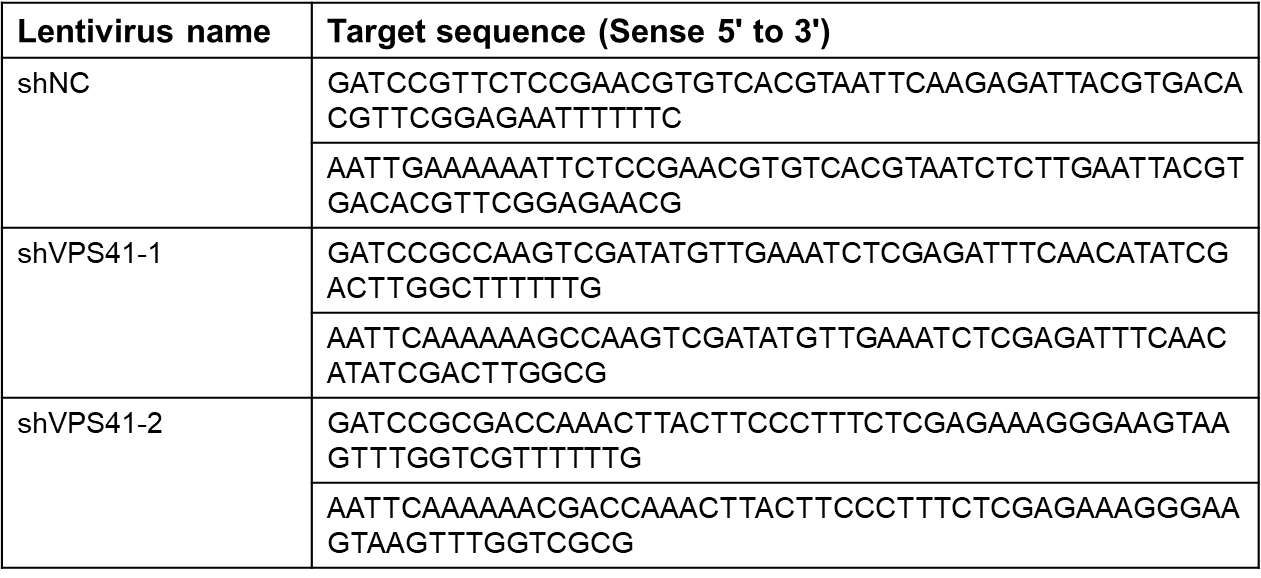
**

**Western blots**

Total protein was isolated using 10% SDS-PAGE and transferred to PVDF membranes. Membranes were blocked with 5% non-fat milk in PBST for 2 hours, then incubated overnight at 4°C with primary antibody diluted in 5% milk in PBST. After five washes with PBST, membranes were incubated with secondary antibody for 2 hours. Protein signals were visualized using an ImageQuant LAS 4000 system (GE Healthcare). Primary antibodies used: anti-VPS41 (1:1000, ab181078, Abcam), anti-RALA (1:1000, ab102555, Abcam), anti-KAI1 (1:1000, #12439, Cell Signaling Technology), anti-Bax (1:1000, ab182733, Abcam), anti-BCL-2 (1:1000, ab32124, Abcam), anti-Caspase 3 (1:2000, #9662, Cell Signaling Technology), anti-c-Caspase 3 (1:1000, #9664, Cell Signaling Technology), anti-PARP (1:1000, #9532, Cell Signaling Technology), anti-c-PARP (1:1000, #5625, Cell Signaling Technology), anti-γH2AX (1:1000, ab81299, Abcam), anti-β-actin (1:10000, ab6276, Abcam), anti-IL-6 (1:1000, #12153, Cell Signaling Technology), anti-TNF-α (1:1000, #6945, Cell Signaling Technology), anti-IFN-β (1:1000, #73671, Cell Signaling Technology), anti-STING (1:1000, #13647, Cell Signaling Technology), anti-IRF3 (1:1000, #4302, Cell Signaling Technology), anti-p-IRF3 (1:1000, #4947, Cell Signaling Technology), anti-GAPDH (1:1000, ab181602, Abcam), and anti-α-Tubulin (1:10000, #2125, Cell Signaling Technology).

**Immunoprecipitation (IP)**

Approximately 10^6^ cells were lysed in buffer for 20 minutes with gentle agitation at 4°C. The lysates were clarified by centrifugation and immunoprecipitated using 1 μg anti-VPS41 (Santa Cruz, CA), anti-Flag (ab205606, Abcam), or anti-HA (ab9110, Abcam) antibodies, incubated overnight at 4°C. Washed protein G-beads (30 μL) were added to the antigen-antibody complexes and incubated for 4 hours at 4°C. The magnetic beads were cleaned four times with lysis buffer, then boiled for 10 minutes with loading buffer. The supernatant was collected by centrifugation at 10,000 rpm for 5 minutes for Western Blot detection.

**Sample preparation for 4D lable free proteomic analyses**

The cellular sample was sonicated three times on ice using a high-intensity ultrasonic processor (Scientz, Ningbo, China) in lysis buffer containing 8 M urea and 1% protease inhibitor cocktail. Residual debris was removed by centrifugation at 12,000 g for 10 minutes at 4°C. The supernatant was collected, and protein concentration was measured using a BCA kit. For trypsin digestion, the protein solution was reduced with 5 mM dithiothreitol for 30 minutes at 56°C, then alkylated with 11 mM iodoacetamide for 15 minutes at room temperature in the dark. The sample was diluted with 100 mM TEAB to reduce urea concentration below 2 M. Trypsin was added at a 1:50 ratio for overnight digestion, followed by a 1:100 ratio for 4 hours. Peptides were desalted using a C18 solid-phase extraction column.

**Sample preparation for mass spectrometry analyses**

After immunoprecipitation of the protein-anti-VPS41-magnetic bead complex, non-specific binding was removed by washing the beads 5 times with pre-cooled PBS. TEAB was added to adjust the pH to alkaline, and the protein solution was extracted by non-contact ultrasonic lysis and quantified using a BCA assay. For trypsin digestion, the protein solution was reduced with 5 mM dithiothreitol for 30 minutes at 56°C, followed by alkylation with 11 mM iodoacetamide for 15 minutes at room temperature in the dark. The sample was diluted with 200 mM TEAB to reduce urea concentration to below 2 M. Trypsin was added at a 1:50 ratio for overnight digestion, followed by a 1:100 ratio for 4 hours. Peptides were desalted using a Strata X solid-phase extraction column.

**Database search for 4D lable free proteomic & mass spectrometry**

MS/MS data were analyzed using the MaxQuant search engine (version 1.6.15.0) by querying the human SwissProt database (20,422 entries) along with a reverse decoy database. Precursor ion mass tolerance was set to 20 ppm for the initial search and 5 ppm for the main search, while fragment ion mass tolerance was set to 0.02 Da. The false discovery rate (FDR) was adjusted to <1%.

**Protein annotation and data analysis**

Protein annotation and functional enrichment analyses were performed as described previously [5]. The Gene Ontology (GO) annotation proteome was retrieved from the UniProt-GOA database ([www.ebi.ac.uk/GOA/](http://www.ebi.ac.uk/GOA/)). Domain functional descriptions were annotated using Pfamscan, which uses protein sequence alignment and the Pfam database (<https://pfam.xfam.org/>). Fisher’s exact test was applied to assess GO category enrichment of differentially expressed proteins against all identified proteins. GO terms with a corrected P-value < 0.05 were considered significant. Protein abundance was assessed using normalized spectral protein intensity. Proteins with Log2 Fold change > 1.2 or < 0.83, and a P-value from the student’s t-test < 0.05, were classified as differentially expressed.

**Combined analysis of 4D proteome and mass spectrometry data**﻿

We aim to identify key proteins interacting with VPS41 and differentially expressed after VPS41 up-regulation through proteomic and mass spectrometry analysis. Differentially expressed proteins in VPS41/Vector (10 Gy) were identified from proteomic data, while interacting proteins of VPS41 (0 Gy and 10 Gy) were extracted from mass spectrometry analyses. The overlap of differentially expressed proteins and VPS41-interacting proteins under 10 Gy irradiation was determined via Venn analysis.

**Inflammation factor protein array**

The Quantibody® Human Inflammation Array-3 chip (RayBiotech, Inc., Norcross, GA) was used to measure cytokine secretion from WS1 cells transfected with VPS41 plasmid and eccDNA following irradiation. WS1 cells were seeded in a 60mm dish and transfected with 2 μg VPS41 plasmid or pcDNA3.1 plasmid and 100ng eccDNA (from irradiated WS1 cells at 10 Gy) once they reached 80% confluency. After 24 hours, cells were irradiated at 10 Gy, and the culture medium was collected 48 hours later. The medium was filtered to 100 μL using an Amicon Ultra-4 filter (Millipore, Bedford, MA). The chip was treated with capture antibodies, followed by biotinylated detection antibodies. Cytokine detection was visualized using streptavidin-conjugated Cy3 dye, and scanning was performed with an InnoScan 300 Microarray Scanner (Innopsys, France) at 532 nm excitation wavelength. Inflammation chip analysis was conducted by RayBiotech Inc.

**Peptide arrays**

For the peptide array, experiments followed the manufacturer’s protocol. An 85 amino acid peptide with a two amino acid offset was synthesized in KAI1-111-228 using an AutoSpot peptide synthesizer and immobilized onto a PEG-modified cellulose membrane to create the peptide array chip. Recombinant VPS41 protein (ReadCrystal, Suzhou, China) was biotinylated using EZ-Link™ NHS-PEG4 (Thermo Fisher, Waltham, MA) and hybridized with the peptide array. The array was incubated with streptavidin-HRP and detected via chemiluminescence, with imaging performed using an imaging device. The preparation and detection were conducted by QYAOBIO (China Peptides Co., Ltd.).

**CCK-8 assay**

In the CCK-8 assay [6], cells were diluted to 1×10^4^/mL and seeded into 96-well plates (100 µL per well) with three replicates per group. After irradiation with 10 Gy, cells were incubated with complete growth medium for 1 to 2 days. CCK-8 solution (Yeasen, Shanghai, China) was added and incubated for 2 hours, then absorbance was measured at 450 nm using a microplate reader.

**LDH assay**

For the LDH assay [7], cells were diluted to 1×10^4^/mL and seeded into 96-well plates (100 µL per well) with three replicates per group. After irradiation with 10 Gy, cells were incubated with fresh culture medium for 1-2 days. LDH Release Assay Kit solution (Beyotime, Shanghai, China) was added and incubated for 30 minutes, followed by absorbance measurement at 490 nm using a microplate reader.

**Colony formation assay**

For the colony formation assay, 3×10^3^ cells were plated in six-well plates with three replicates per group in complete growth medium. About ten days post-irradiation, cells were fixed with methanol, stained with 0.05% crystal violet, and photographed.

**Cell apoptosis detection**

Cell apoptosis was detected using the Annexin V-FITC apoptosis detection kits (Yeasen, Shanghai, China) [8]. Briefly, 1 × 10^5^ cells were resuspended in 400 μL binding buffer, labeled with Annexin V-FITC (5 μL) and propidium iodide (PI) (10 μL) for 15 minutes in the dark at room temperature. Green fluorescence (Annexin V-FITC) and red fluorescence (PI) were analyzed using flow cytometry (MilliporeSigma Co., Ltd., Burlington, VE) with an excitation wavelength of 488 nm and emission wavelength at 530 nm.

**ROS generation assay**

Cells were seeded in 10 cm dishes and irradiated as specified [9]. Adherent cells were treated with 5 μM CM-H2DCFDA (Beyotime, Shanghai, China) for 30 minutes at 37 °C, then washed with PBS. Stained cells were trypsinized, resuspended in PBS, and ROS generation was evaluated using flow cytometry (excitation at 488 nm, emission between 515 and 545 nm) with 2 × 10^4^ cells per condition.

**Confocal microscopy imaging**

For the immunofluorescence assay, cells were rinsed with PBS, fixed with 4% formaldehyde, and blocked with 2% BSA in PBS for 1 hour at room temperature. Primary antibodies targeting γH2AX (ab81299, Abcam) were incubated overnight at 4 °C, followed by secondary antibodies conjugated with Cy3 (Beyotime, Shanghai, China) for 1 hour at room temperature. DAPI (Invitrogen, Carlsbad, CA) was used to label the nuclei, and images were captured using the Olympus FV1000 microscope (Olympus, Japan). For co-localization analysis, cells transfected with VPS41-EGFP plasmid were stained with lysosomal and mitochondrial dyes (A66443, Thermo Fisher Scientific) and analyzed by confocal microscopy. To assess co-localization of truncated VPS41-EGFP constructs with KAI1-BFP and lysosomes (LysoTracker, Yeasen), cells co-transfected with VPS41-EGFP and KAI1-BFP plasmids were analyzed 24 hours post-transfection. Co-localization was analyzed using the ImageJ "colocalization" module.

**Electron microscopy**

HaCaT cells were fixed in 4% glutaraldehyde at 4 °C for 2 hours, rinsed three times with 0.1 M sodium dimethyl arsenate, and postfixated with 1% osmium tetroxide at 4 °C for 90 minutes. After three washes with distilled water, dehydration was performed through ethanol and acetone solutions, followed by two rounds of 100% propionaldehyde dehydration. The samples were embedded in media at ratios of 1:3, 1:1, and 3:1, and polymerized at 35 °C for 24 hours, 45 °C for 24 hours, and 60 °C for 24 hours. Ultrathin sections (70-90 nm) were stained with lead citrate and uranyl acetate and examined under an electron microscope (H-7650; Hitachi, Tokyo).

**AlphaFold 3-based prediction of protein interaction structure**

Amino acid sequences were uploaded to the AlphaFold 3 platform for structure prediction, generating multiple potential conformations. The conformation with the highest confidence was selected for analysis. Structural data in CIF format was downloaded and visualized using PyMOL software. Hydrogen bond interactions between the two proteins were analyzed, and key interaction sites were annotated, providing essential structural information for subsequent biochemical experiments.

**Hematoxylin and eosin (H&E) staining**

Skin specimens were fixed in 10% neutral-buffered formalin and embedded in paraffin. Three-micrometer sections were deparaffinized and treated with citrate buffer (pH 6.0) for 7 minutes for epitope retrieval. The sections were then stained with hematoxylin and eosin (H&E) (ZSGB-Bio, Beijing, China).

**Immunohistochemical staining**

Tissues were fixed in 10% neutral buffered formalin and embedded in paraffin. Three-micrometer sections were deparaffinized, treated with citrate buffer (pH 6.0) for 7 minutes for epitope retrieval, and incubated with 3% hydrogen peroxide for 15 minutes to block endogenous peroxidase activity. Non-specific binding was blocked with 4% skim milk for 30 minutes. Sections were incubated with anti-VPS41 antibody (GTX89439, GeneTex) at a 1:200 dilution for 1 hour, followed by incubation with an HRP-conjugated secondary antibody for 30 minutes. Color development was achieved using DAB, and counterstaining was performed with hematoxylin. All steps were conducted at room temperature. Human skin tissue samples for IHC detection were collected at the Nuclear Industry 416 Hospital (Chengdu, China). Informed consent for sample collection was obtained from all participating patients. The detection of human skin tissue was approved by the Ethics Committee of the Nuclear Industry 416 Hospital (Chengdu, China).

**Supplemental results**


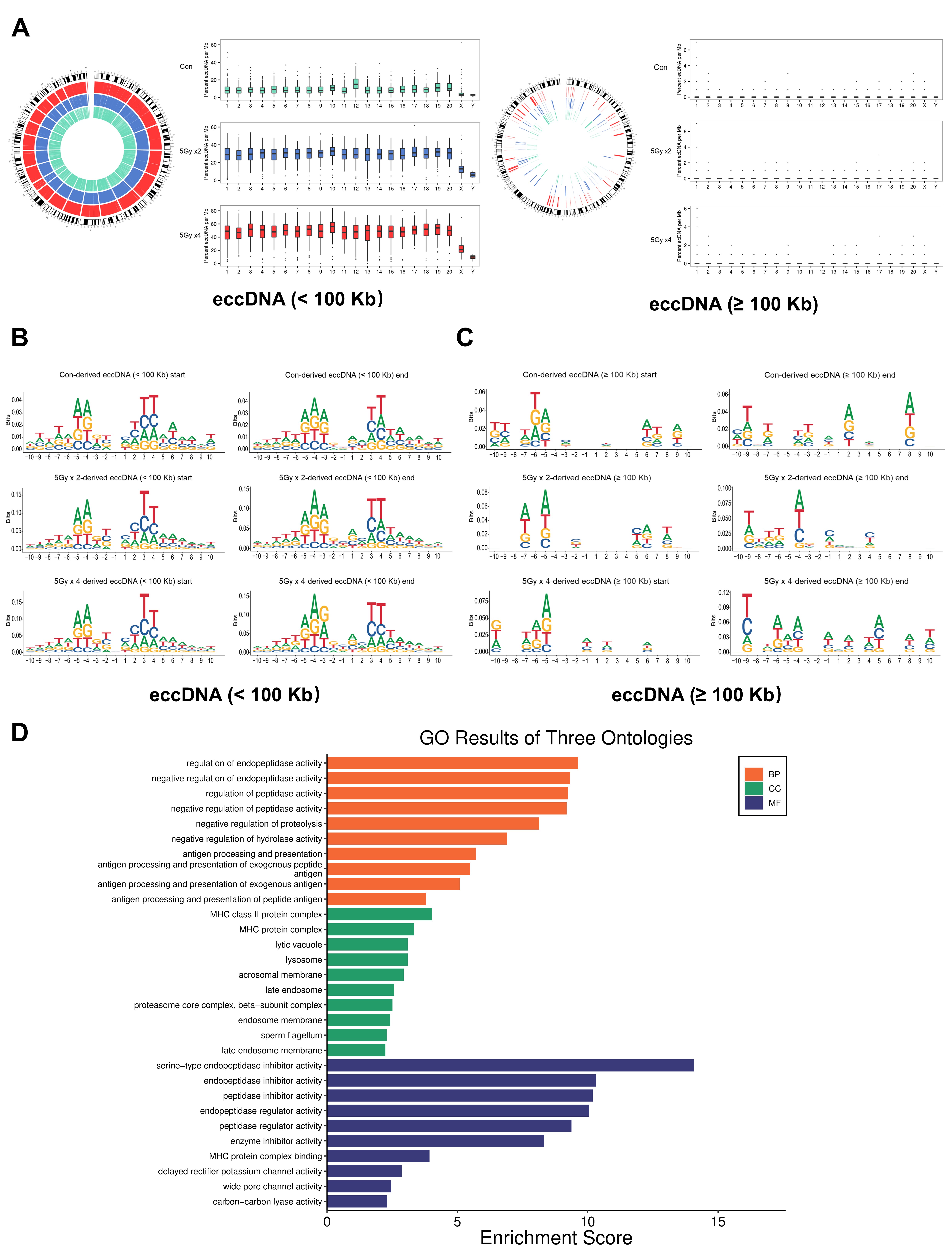


**Supplementary Figure 1. Information of total eccDNA.** (A) Frequency distribution of eccDNA across chromosomes. (B) Sequence analysis of junction sites for eccDNA (< 100 Kb) (within 10 bp range). (C) Sequence analysis of junction sites for eccDNA (≥ 100 Kb) (within 10 bp range). (D) GO enrichment analysis of eccDNA (≥ 100 Kb) (n=19) with 150 changed genes and split reads ≥ 20.


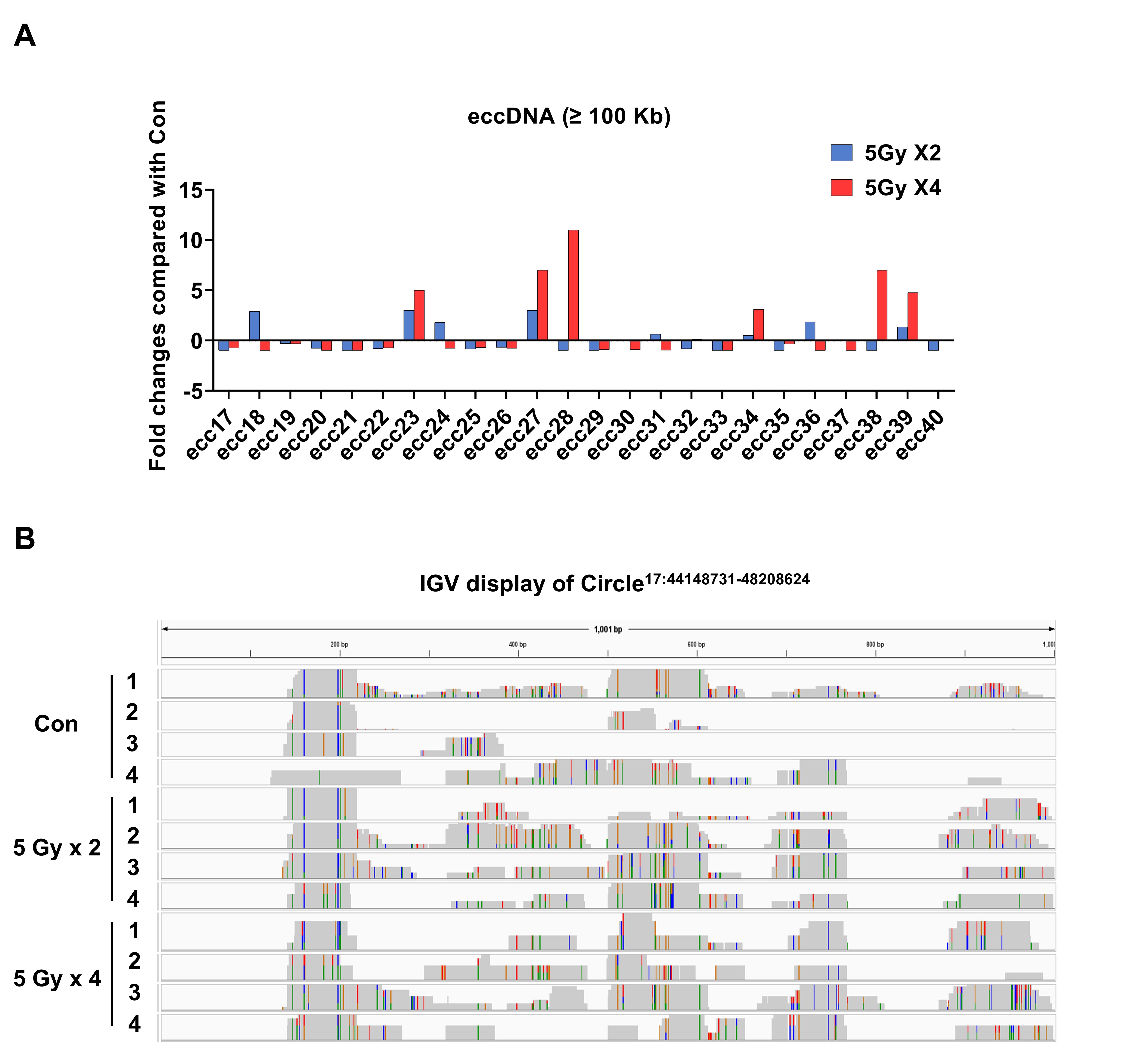


**Supplementary Figure 2. Information of eccDNA (≥ 100 Kb)** **for verifying.** (A) Fold change data for eccDNAs with both split and discordant reads reaching 10. (B) IGV visualization of circle^17:44148731-48208624^.





**Supplementary Figure 3. Validation of junction sequences for other candidates of eccDNAs (< 100 Kb).** (A, E-H) Negative results from the gel electrophoresis of PCR products for the junction sequences of ecc1 and ecc6-ecc8 are illustrated. (B-D) The IGV results for ecc2-ecc4 showed relatively low genomic coverage or lengths that were too short to warrant validation.





**Supplementary Figure 4. Validation of junction sequences for other candidate eccDNAs (≥ 100 Kb).** (A, C-H) The gel electrophoresis from PCR products showed negative results for the junction sequences of ecc17, ecc18, ecc29, ecc31, ecc34, ecc27, ecc37, and ecc39. (B) The IGV result of ecc18 indicate relatively low genome coverage or insufficient length, and further validation is not considered.


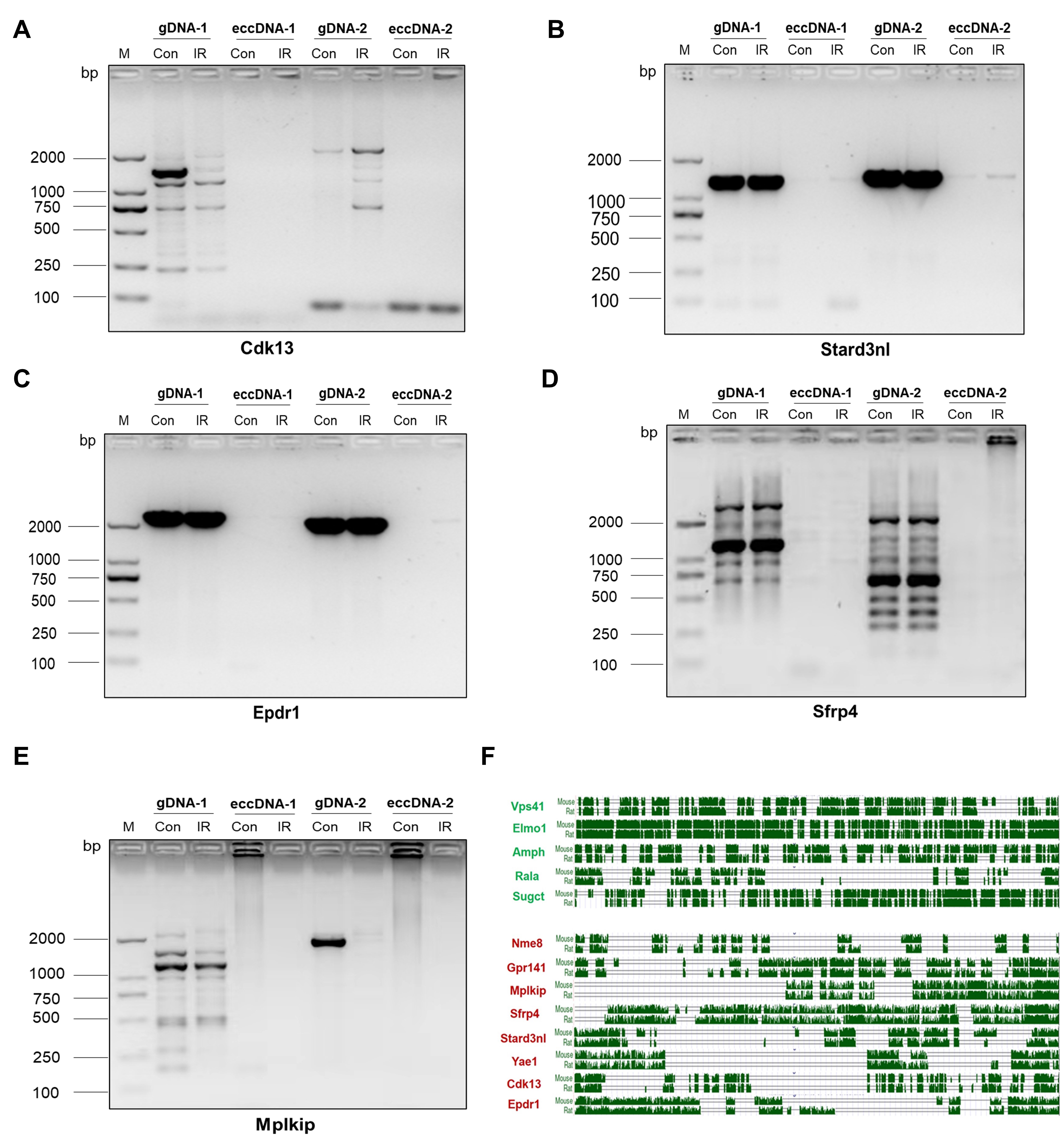


**Supplementary Figure 5. PCR analysis of additional genes on circle****^17:44148731-48208624^.** (A-E) Negative PCR results for Cdk13, Stard3nl, Epdr1, Sfrp4, and Mplkip. (F) Compare the sequence conservation of selected (green font) and non-selected genes (red font) in eccDNA using UCSC Genome Browser data (human GRCh38/hg38).





**Supplementary Figure 6. Protective effect of eccDNA and VPS41.** (A) HE staining of irradiated rat skin (45 Gy, 65 days, and scale bar: 100 μm). (B) Workflow for inflammatory factor detection 48 hours post-irradiation (10 Gy) in eccDNA-transfected WS1 cells (n=5 per group). (C) GO analysis of secretory inflammatory factors in eccDNA-transfected WS1 cells. (D) PCR analysis of eccDNA^VPS41^ amplification in WS1 cells at varying irradiation doses. (E) Western blot analysis of inflammatory factors (TNF-α, IL-6, IL-1, IFN-γ) in WS1 cells 48 hours post-eccDNA transfection. (F) Western blot analysis of DNA damage inducers on the expression of γ-H2AX. DNA damage treatments for 2 hours include ionizing radiation (6 Gy), UVB (20 mJ/cm²), etoposide (2 μM), paclitaxel (20 nM), and cisplatin (2 μM). (G) Apoptosis assays of VPS41 upregulation after UVB (10 mJ/cm2) exposure for 48 hours. (H) Apoptosis assays of VPS41 upregulation after paclitaxel (20 nM) treatment for 48 hours. *P* values were determined using appropriate statistical methods according to the analysis type: Mann–Whitney U test for two-group comparisons and Fisher’s exact test for GO enrichment analysis. Statistically significant differences are denoted as follows: **P* < 0.05, ***P* < 0.01. Data are presented as mean ± SD (n = 3) unless otherwise specified.


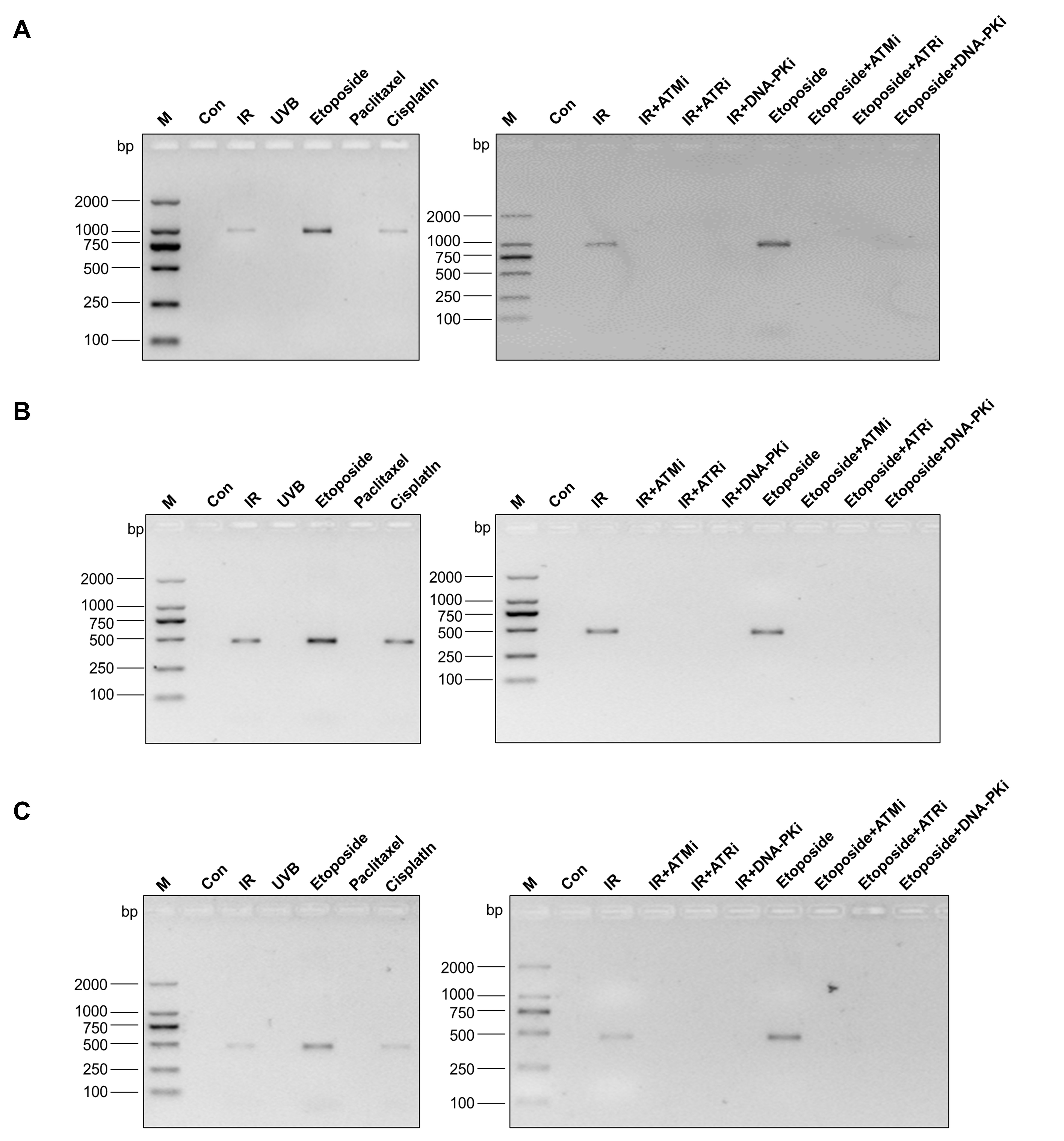


**Supplementary Figure 7. Impact of DNA damage inducers and inhibitors on eccDNA^VPS41^ formation assessed by PCR analysis.** HaCaT cells are used to amplify VPS41 from exon 29 of circle^7:38722974-38276326^. DNA damage treatments include ionizing radiation (6 Gy), UVB (20 mJ/cm²), etoposide (2 μM), paclitaxel (20 nM), and cisplatin (2 μM). DNA damage inhibitors include 2 μM ATMi (KU-55933), 2 μM ATRi (AZD6738), and 2 μM DNK-PKi (AZD-7648). (A) Promoter region upstream 2000 bp. (B) Middle region of exon 29. (C) 3' terminal region of exon 29.





**Supplementary Figure 8. Impact of DNA damage inducers and inhibitors on the STING pathway.** The selected DNA damage treatments include IR at 6 Gy, UVB exposure at 20 mJ/cm², etoposide at 2 μM, paclitaxel at 20 nM, and cisplatin at 2 μM. The chosen DNA damage inhibitors comprise 2 μM ATMi (KU-55933), 2 μM ATRi (AZD6738), and 2 μM DNK-PKi (AZD-7648). (A-D) DNA damage pre-treatments were conducted for 1, 2, 4, and 24 hours. (E-H) Three DNA damage inhibitors were subjected to pre-treatment for 12 hours, followed by IR (6 Gy) and etoposide (2 μM) pre-treatments for 1, 2, 4, and 24 hours.





**Supplementary Figure 9. Impact of DNA damage inducers on VPS41 expression.** The selected DNA damage inducers include IR (2 Gy and 10 Gy), UVB (5mJ/cm^2^ and 10mJ/cm^2^, etoposide (2 μM and 10 μM), cisplatin (2 μM and 10 μM) and paclitaxel (2 μM and 10 μM). Cell lysates were collected 48 hours after IR and UVB, and 48 hours after 12-hour pre-treatment with other inducers. (A-B) HaCaT. (C-G) HEK-293T. (H-L) A375. (M-Q) SiHa.


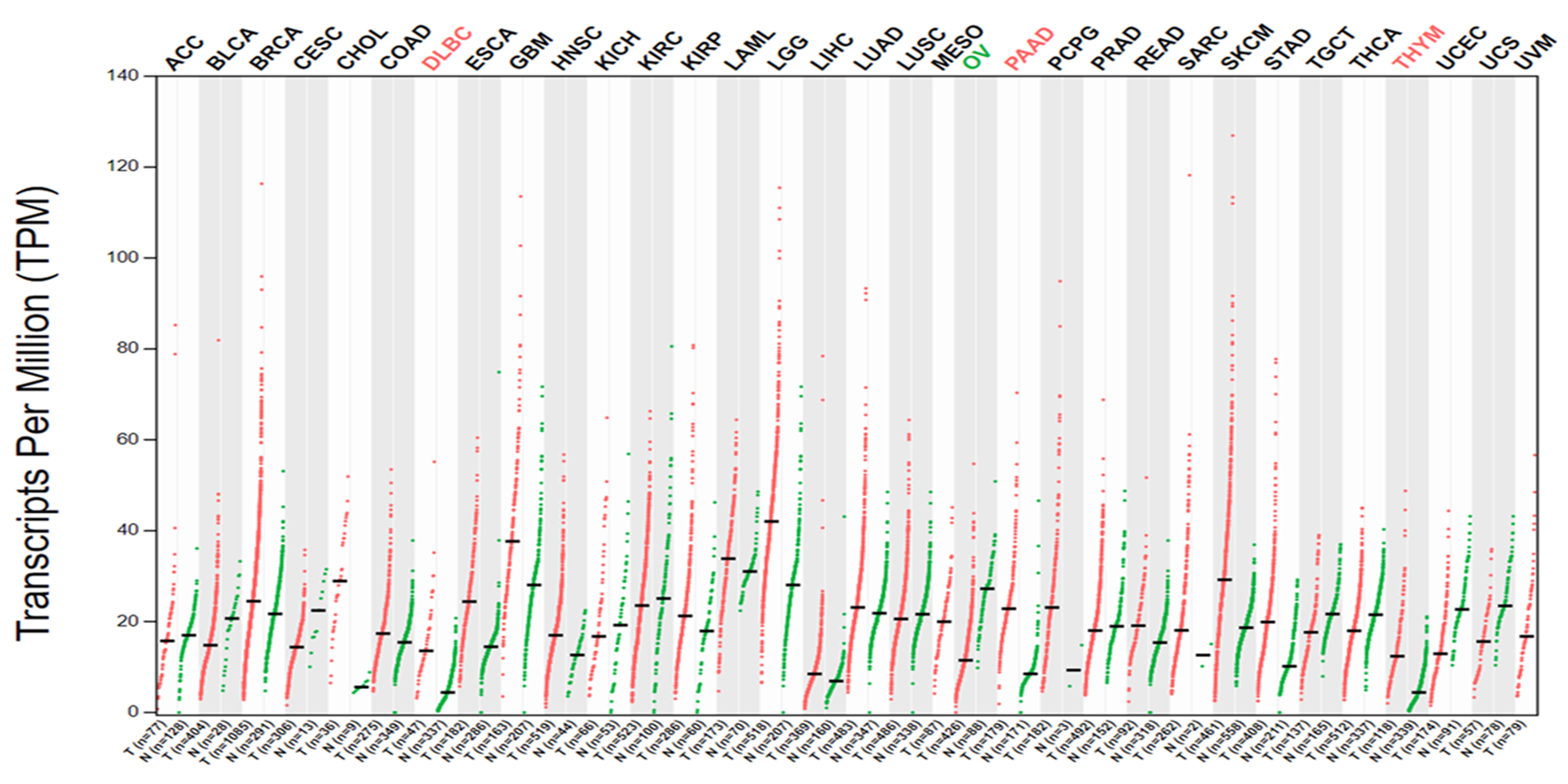


**Supplementary Figure 10. VPS41 expression in tumor patients vs. healthy individuals.** GEPIA 2 analysis shows no significant difference in VPS41 expression between tumor tissues (red dots) and healthy controls (green dots) in most cancer patients. *P* values were determined with a t test using the median of Log_2_ Fold Change in each group. Statistically significant differences are indicated by *P* < 0.05, with red tumor names representing upregulation, green tumor names representing downregulation, and black tumor names indicating no significant change.


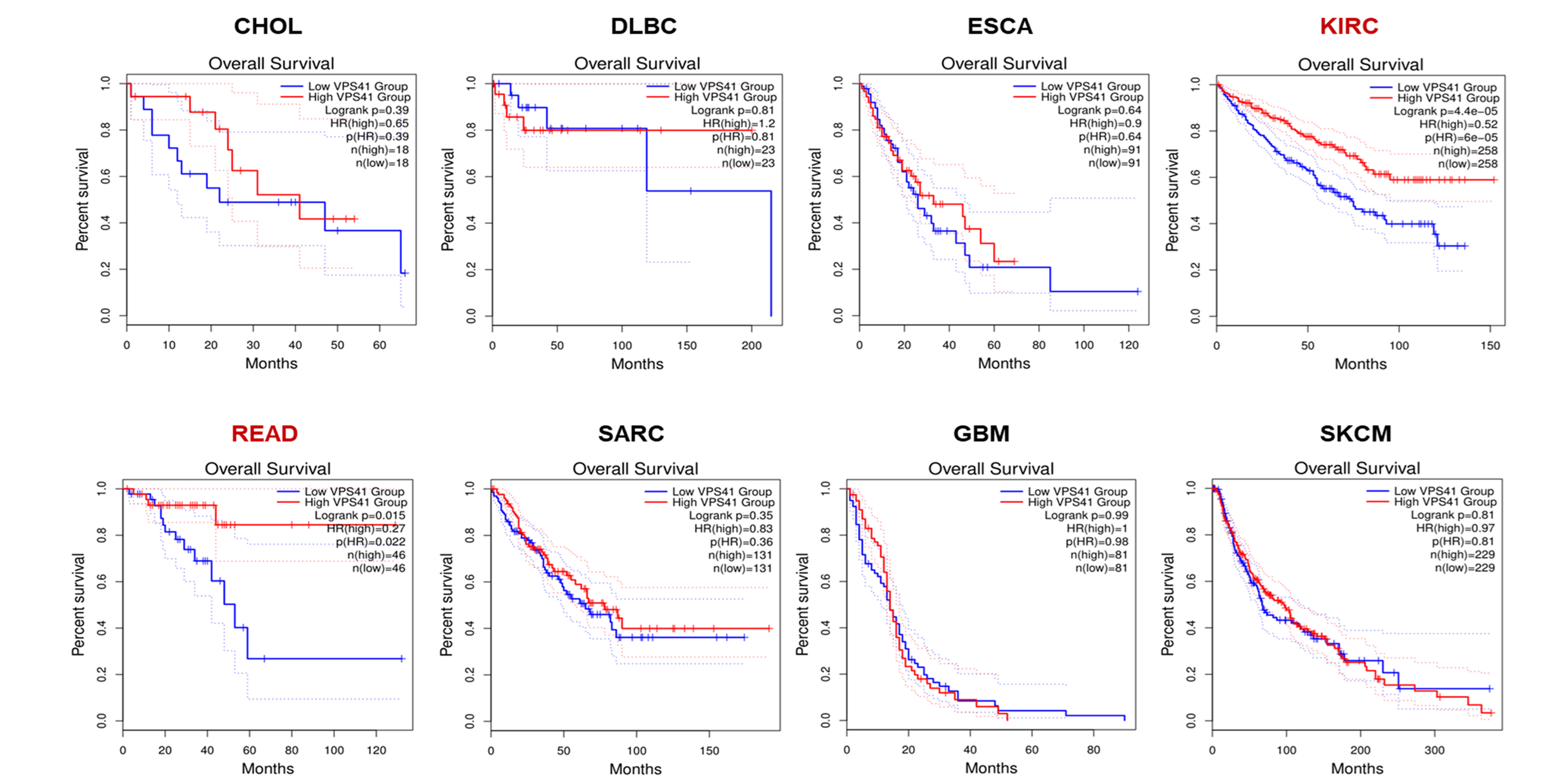


**Supplementary Figure 11. The** **correlation between VPS41 expression and survival rate of tumor patients.** GEPIA 2 analysis reveals a correlation between VPS41 expression and patient survival across tumor types. Elevated VPS41 expression in tumor tissues is associated with a favorable prognosis in KIRC and READ. *P* values were determined with a Log-rank test. Statistically significant differences are indicated by *P* < 0.05, with red line representing upregulation, bule line representing downregulation.





**Supplementary Figure 12. Radioprotective effects of VPS41 and KAI1 truncated variants.** (A) KAI1 mRNA expression in HEK-293T cells. (B) KAI1 protein levels in WS1, HaCaT, and HEK-293T cells. (C) Apoptosis assay assessing the effect of VPS41 truncations on radiation-induced apoptosis in HaCaT cells. (D) Apoptosis assay evaluating the effect of KAI1 truncations on radiation-induced apoptosis in HaCaT cells. *P* values were calculated using one-way ANOVA followed by Bonferroni’s post hoc test for multi-group comparisons. Statistically significant differences are denoted as follows: **P* < 0.05, ***P* < 0.01. Data are presented as mean ± SD (n = 3) unless otherwise specified.


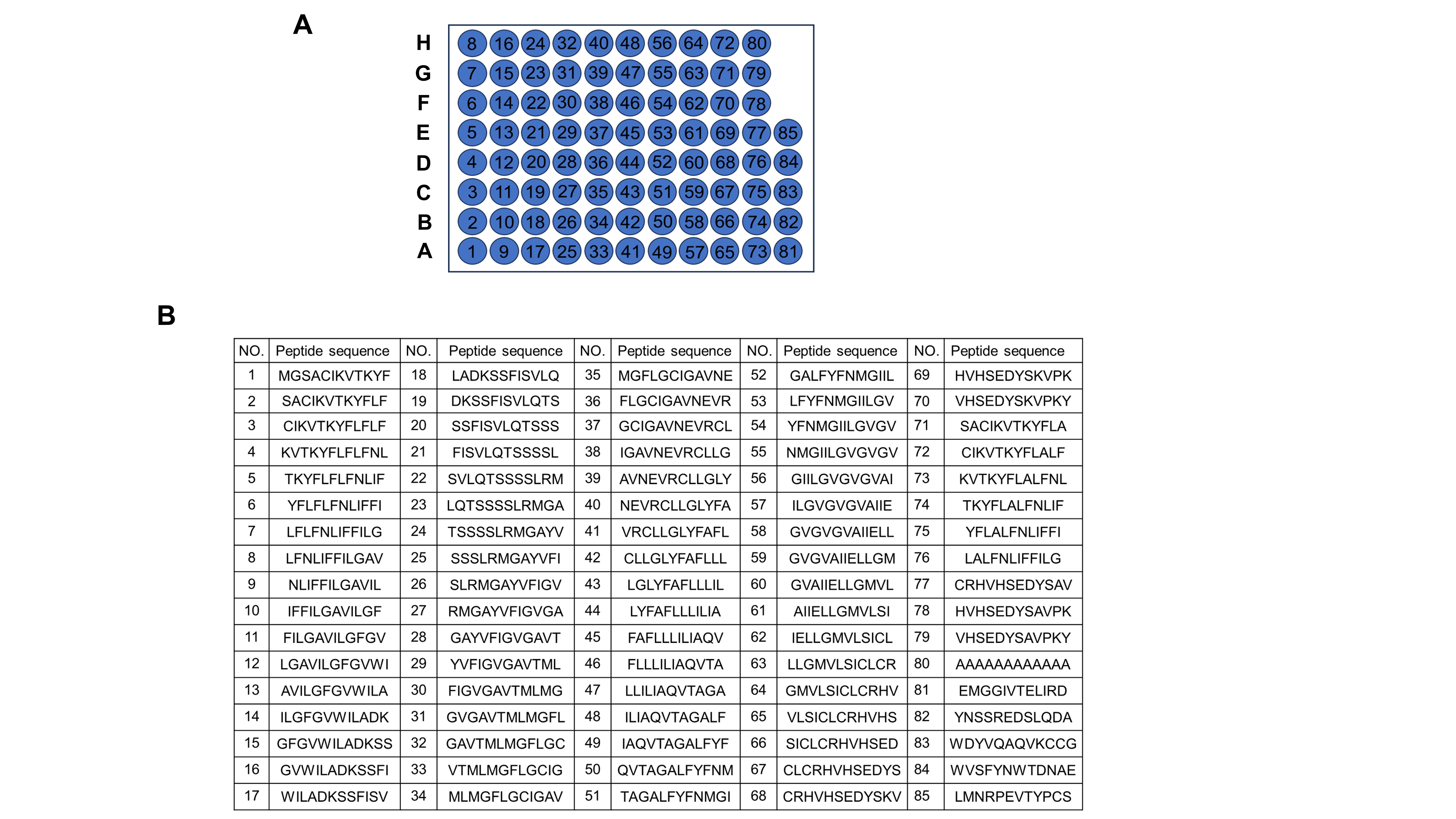


**Supplementary Figure 13. Synthetic peptide distribution of polypeptide arrays.** (A) Dot matrix diagram of peptide sequences. (B) List of synthesized peptide sequences.

**Supplementary Table 1. Counts of eccDNA (< 100 Kb) and eccDNA (≥ 100 Kb) in** **each sample.**

| Identifier | eccDNA (< 100 Kb) | eccDNA (≥ 100 Kb) |
| --- | --- | --- |
| Con-1 | 4804 | 35 |
| Con-2 | 9503 | 71 |
| Con-3 | 10441 | 72 |
| Con-4 | 3643 | 64 |
| 5 Gy x 2-1 | 19044 | 73 |
| 5 Gy x 2-2 | 28769 | 59 |
| 5 Gy x 2-3 | 27452 | 32 |
| 5 Gy x 2-4 | 6347 | 35 |
| 5 Gy x 4-1 | 22122 | 35 |
| 5 Gy x 4-2 | 44724 | 49 |
| 5 Gy x 4-3 | 46187 | 52 |
| 5 Gy x 4-4 | 20148 | 56 |

**Supplementary Table 2. Information of candidate eccDNA (< 100 Kb).**

| **Identifier** | **Region** | **Spilt reads** | **Discordant reads** | | **Length(bp)** | **Position information** |
| --- | --- | --- | --- | --- | --- | --- |
| **ecc1** | intron | 60 | | 63 | 653 | Chr.20: 48001335-48001988 |
| **ecc2** | intron | 54 | | 71 | 879 | Chr.20: 48056410-48057289 |
| **ecc3** | intron | 16 | | 170 | 663 | Chr.20: 48071367-48072030 |
| **ecc4** | intron | 40 | | 642 | 895 | Chr.20: 48137161-48138056 |
| **ecc5** | intron | 86 | | 17 | 234 | Chr.20: 48144968-48145202 |
| **ecc6** | intron | 36 | | 128 | 778 | Chr.16: 83836517-83837295 |
| **ecc7** | intron | 17 | | 1093 | 2,790 | Chr.16: 83053397-83056187 |
| **ecc8** | intron | 20 | | 74 | 609 | Chr.1: 81872754-81873363 |
| **ecc9** | intron | 37 | | 650 | 1,566 | Chr.2: 240801712-240803278 |
| **ecc10** | intron | 29 | | 155 | 184 | Chr.6: 12841340-12841524 |
| **ecc11** | intron | 135 | | 6 | 258 | Chr.12: 49704824-49705082 |
| **ecc12** | intron | 11 | | 136 | 821 | Chr.9: 118861031-118861852 |
| **ecc13** | intron | 8 | | 107 | 487 | Chr.15: 55175166-55175653 |
| **ecc14** | intron | 95 | | 477 | 1,173 | Chr.13: 70748379-70749552 |
| **ecc15** | intron | 8 | | 174 | 358 | Chr.10: 87847149-87847507 |
| **ecc16** | intergenic | 39 | | 1236 | 11429 | Chr.9: 40512521-40523950 |

**Supplementary Table 3. Information of candidate eccDNA (≥ 100 Kb).**

| Identifier | Region | Spilt reads | | | Discordant reads | Length(bp) | Position information |
| --- | --- | --- | --- | --- | --- | --- | --- |
| ecc17 | exon | | 85 | 40 | | 932,399 | Chr1:15315244-16247643 |
| ecc18 | exon | | 60 | 117 | | 104,297 | Chr1:24110808-24215105 |
| ecc19 | exon | | 30 | 358 | | 342,580 | Chr1: 55683015-56025595 |
| ecc20 | exon | | 16 | 74 | | 1,764,903 | Chr13: 89439405-91204308 |
| ecc21 | exon | | 19 | 41 | | 349,135 | Chr14: 94273598-94622733 |
| ecc22 | exon | | 11 | 52 | | 125,411 | Chr15: 5420892-5546303 |
| ecc23 | exon | | 24 | 51 | | 4,059,893 | Chr17: 44148731-48208624 |
| ecc24 | exon | | 11 | 540 | | 259,662 | Chr17: 44229313-44488975 |
| ecc25 | exon | | 10 | 892 | | 126,840 | Chr17: 44298077-44424917 |
| ecc26 | exon | | 19 | 58 | | 279,208 | Chr17: 71781078-72060286 |
| ecc27 | exon | | 16 | 33 | | 6,900,317 | Chr18: 57670976-64571293 |
| ecc28 | exon | | 12 | 90 | | 4,727,675 | Chr19: 47911191-52638866 |
| ecc29 | exon | | 42 | 1806 | | 641,755 | Chr.2: 79795510-80437265 |
| ecc30 | exon | | 54 | 4502 | | 2,014,610 | Chr.2: 159003947-161018557 |
| ecc31 | exon | | 13 | 1521 | | 994,374 | Chr.2: 260386379-261380753 |
| ecc32 | exon | | 42 | 267 | | 166,887 | Chr.20: 4628564-4795451 |
| ecc33 | exon | | 23 | 34 | | 2,641,978 | Chr.20: 28384188-31026166 |
| ecc34 | exon | | 183 | 77 | | 786,602 | Chr.20: 46561281-47347883 |
| ecc35 | exon | | 43 | 28 | | 1,071,350 | Chr.20: 48947652-50019002 |
| ecc36 | exon | | 80 | 29 | | 511,300 | Chr.5: 148172368-148683668 |
| ecc37 | exon | | 54 | 243 | | 350,200 | Chr.6: 10465475-10815675 |
| ecc38 | exon | | 12 | 18 | | 994,374 | Chr6: 44762751-45873807 |
| ecc39 | exon | | 51 | 1639 | | 4,185,653 | Chr9: 8276021-12461674 |
| ecc40 | exon | | 38 | 84 | | 350,200 | ChrX: 17880835-18250828 |

**Supplementary Table 4. PCR primers information of candidate eccDNAs.**

| Identifier | Forward | Reverse |
| --- | --- | --- |
| ecc1-1 | CCGCGAGCACAGGGAGCACCGCAAG | GCTCACTGTGCTCCCTGTGCTGTGC |
| ecc1-2 | GCACAGCACAGCACAGCGAGCACCG | GCTCACTGTGCTCCCTGTGCTGTGC |
| ecc1-3 | AGGAAGCACTGCGAGCACAGGGAGC | CTGTGCTCGTTGTGCTGTGCTCGCT |
| ecc5-1 | ACACACAAACACACAAAAGCATGTG | TGTATGTGTGCATGTTTTCATGTGTGT |
| ecc5-2 | GCATGCACACACACACAAACACACA | TGTGTGCACATGCTTTTGTGTGTTTT |
| ecc5-3 | ATGAAAACATGCACACATACACATTAAAAACAC | ACATGCTTTTGTGTGTTTGTGTTTGTGTGG |
| ecc6-1 | AGACACACACACACACAGACACACAG | GTATATGTATGTGTGTGTATGTGTGTGTGT |
| ecc6-2 | ACATATACACACACACACACAGACAC | TACTGTGTGTGTGTGTCTGTGTGTG |
| ecc6-3 | ACAGAGACACACACACTCACAGACA | TGTCTGTGTGTGTGAGTGTGTACTG |
| ecc6-4 | CAGACACACACACACAGACACACAT | TGTGTGTGTGTGTGTGTGTGTGTGC |
| ecc6-5 | CAGACACACACACACAGACACACAT | TGTGTGTGTGTGTGTGTGTGTGTGT |
| ecc6-6 | GACACACACACTCACAGACACACAT | TGTGTGTGTGTGTGTGTGTGTGTGC |
| ecc6-7 | CAGACACACACACACAGACACACAT | TGTGTGTATATGTATGTGTGTGTATGTGTGT |
| ecc7-1 | CTGTACTCTGACACTGTACTCTGGGAC | GAGTACAGTGTCATACAGTACAGTGTCCCAGAGT |
| ecc7-2 | GTGCTCTGTGACACTGTACTCTGGGA | GAGTACAGTGTCATACAGTACAGTGTCCCAGAGT |
| ecc7-3 | GCACTGTACTCTGTGACACTGTGCT | CTCCAGAGCACAGTGTCACAGAGCA |
| ecc7-4 | GTACTCTGGGGCACTGCACTGTACT | TCATACAGTACAGTGTCCCAGAGTAC |
| ecc8-1 | TTGCCAGCCTAGCTGCTGTGAAACC | CAGCTGGTCACACAGGCTGGTTTGC |
| ecc8-2 | GCTGTGAAACCACCTGTTGTAAGAC | AGCAGGTGGTTTCACAGCAGATTGG |
| ecc15 | ACCAGCTGCTGCAGCCCAATCTGCT | CAGCTGGTCACACAGGCTGGTTTGC |
| ecc16 | CTCTCTCCCTGTCTCTCTATGTCTCTG | CAGGGAGACAGGGAAACAGAGAGCA |
| ecc17-1 | CTCACACACAGCACTCACACACAGCT | TGTGTGTGAGTGCTGTGTGTGAGAG |
| ecc17-2 | GCACTCACACACAGCTTTCAGACAG | TGTGTGTGAGTGCTGTGTGTGAGAG |
| ecc17-3 | CTCACACACAGCACTCACACACAGCT | TGCTGTGTGAGTACTGTCTATGAGTG |
| ecc23 | GGTGAATTTGGTAGGATCCCAAGAG | ACAAGAGCCTGATAATGGGAAGTAAATGAA |
| ecc27-1 | TCTAAGGTTCTTCCTCACAGTGCTG | CCAGTGGTCCTCAACCTTCCTAATG |
| ecc27-2 | TTTAAGCATGATGGCCAGAGTCATAAAC | TCCAAGCCATAAAATTATTTTTGTTGCTACCT |
| ecc27-3 | GGCCAGAGTCATAAACATACAGACC | ATCATAATGTAGACCTGTGTTTTCTGATGGT |
| ecc29-1 | TATGTGGGACACTGAGTCTGTGCTC | CAGAGTGTCCCACAAGCAGAGCACA |
| ecc29-2 | CGTAGGACACTGTGTCTGTGCTCTC | CAGAGTGTCCCACAAGCAGAGCACA |
| ecc29-3 | CACTGTGTCTGTGATCTGCACGTAG | CAGAGTGTCCCACAAGCAGAGCACA |
| ecc29-4 | TTTGTGCTCTGCATGTGGGACACTG | ATGCAGAGAACACAGACCCAATGTC |
| ecc31-1 | GGTAGTGAGCTCATAGTGATGCTCA | TCCACACGTTGTCACGGTCACTGTG |
| ecc31-2 | GTGAGCTCACAGAGACGCTCACGTG | TCCACACGTTGTCACGGTCACTGTG |
| ecc31-3 | GTGAGCTCACAGAGACGCTCACGTG | ACGTGAGCATCACTGTGAGCTCCCT |
| ecc31-4 | AGAGACGCTCACGTGGATGGTAGTG | TCCCTACCATCCATGTGAGCATCAC |
| ecc34 | GGGGCCCACACAGGGTGGGCACCTG | AGTGCTCCACCCTGTGTGAGTCCTT |
| ecc37-1 | CATGGGGCCTAGGTGTGCCGTGGGG | GGCACATGTAGGCCCCACGGCACAC |
| ecc37-2 | GTGTGCCGTGGGGCCTAGGTGTGCT | ATGTAGGCCCCACGGCACACCTAGG |
| ecc37-3 | CCGTGGGGCCTAGGTGTGCTGTGAG | GGGCCCCACGGCACACCTAGGCCTC |
| ecc39-1 | ATAAGTGAAATTGCCTATAGGCAAATCCAG | TTCTTTGTGACCACTCTCCACATACTAC |
| ecc39-2 | ATGTTCAGTCCTTGCACTTTTGTAAATGAT | ATAATTGAGTTGGGGATAGTATTCACCATAGC |
| ecc39-3 | ATGTTCAGTCCTTGCACTTTTGTAAATGAT | CACATAAAACACACACACTCACACACAT |
| ecc39-4 | CTCTTAGGATGGGCTCTAATTTGTAGATTCC | TCACATACATACACACAGACACACACAC |

**Supplementary Table 5. PCR primers information of rat genes from circle^17:44148731-48208624^.**

| Gene | Forward | Reverse |
| --- | --- | --- |
| Mplkip-1 | GACTCGCTAAAGTCCAGGACTCTGG | CGTGGTACACACTCCCAGATAGCTA |
| Mplkip-2 | TCTTCCGCTTCCGTTTCCGTATACT | CAAGATGTTCCTGACACATGAGGCT |
| Cdk13-1 | TCTGCGCACACACGGCGACTAGAAA | CTGAGTACTGGACTGTAGGGACTGC |
| Cdk13-2 | GGCAAGATGACCTCATCCAACACCA | AATGACCTAATAGCACTGCTCTAATCTGG |
| Rala-1 | TTTGTAGAAGACTATGAACCTACCAAAGCAG | CTGAAGTCCGCTGTAGCTGCAAAGG |
| Rala-2 | AAGCATTGCCATTGGAGGCTCAATT | GCAGATTCACAGATGAATTACTCTCAGTT |
| Sugct-1 | GATGGAGATCCTGTTCGCCCAGGAG | CCAGTTCTGTAACGTTGTATGAGGC |
| Sugct-2 | GTCCAGCTGTGAGATACAGCAAGTT | TAATCTTCAGTTCTCCTCACCATGCT |
| Vps41-1 | GAAAGAGAATACTGTGCAAGACCGAG | CTAGGTGGTAATCCCTACATTCATTCTCCT |
| Vps41-2 | AATGCTCCTGCACAGTACTGTAACA | ATGCATAGCATGTACGTAGCAAGCA |
| Amph-1 | CTCCGCTCTATCTCTTTAAGCCGGT | CGAAGATGCCCGTCTTGATGTCGGC |
| Amph-2 | ATCCCTTCTGTTGTCATAGAGCCAG | CTTGTAGAGAAAGCCAGGAGGCAGT |
| Stard3nl-1 | GTTGCTTCTCACTAGGGATGGTGGA | ACGTTACAAATAAGAGGTCAAAGGTGACA |
| Stard3nl-2 | GACTACTGCTAGTTCAGGATGCATCG | TTACTGTGTGACGCTGGTGTACTTC |
| Epdr1-1 | AAGGATTGCTACCCGGTCCAGGAGA | TTGCATTAGTACAAGTCTCCCTAGCAAC |
| Epdr1-2 | GGATTGGCGTTTATACAGCCAAGGA | GATGGCTACAGCATAAAGCAAGCTT |
| Nme8-1 | GGTGCAAATGCACCACTTATCAATAG | CTTCGTCCAATGAGTCTACTAGTGGAA |
| Nme8-2 | CACTATATTGATGCAGAGGCAAGTCG | CTGTCTGGATGAGATGCATAAGCCT |
| Sfrp4-1 | GTTCAGTGCGGACTGGAGCTCCAGC | CTGTCTGGATGAGATGCATAAGCCT |
| Sfrp4-2 | CAGAACTCCCGTTCAGTGCGGACTG | AACAGCAGAGAACAACTCAGGACAA |
| Gpr141-1 | ACCTCGGAGAATTCCTCTTGTGACC | AACAGTCATGTTTGCAGTGTGTTACTAC |
| Gpr141-2 | AGTAGGCGTCATCTCCATCCTGTTC | CTTCCAGGAATAATTGCTCTCTTGACCC |
| Elmo1-1 | GTCGTGGGTACCTGGTCCTAGGCAC | CCTCTATCTCGTGCCCATCCTCTCC |
| Elmo1-2 | CTCAGAAGCAGCTGCGCTACATCAT | TGGTCATCATCCTATCTTCCAGAAGG |

**Supplementary Table 6. Other primers information.**

| Gene | Forward | Reverse |  |
| --- | --- | --- | --- |
| VPS41- promoter | TAATTCTGCTCCTGTGCTCCTATCC | GCTGCTCCATGTATAGGATGCAGAA | |
| VPS41- 5' terminal | GGGACCACCTGATGCTGAA | GCCCTGCTCTGAAGTTAGGC | |
| VPS41- middle | ATCCTGCAAGCTGCTACCTG | TCCTTCAGCATCAGGTGGTC | |
| VPS41- 3' terminal | CAGTGCTAAGAACCGTGGAC | GCAAGAATCGTAGCCAGCA | |
| KAI1-1 | ATCTTCTTTATCCTGGGCGCAGTGA | GTTGTCTGTCCAGTTGTAGAAGCTG | |
| KAI1-2 | CTTCTACTTCAACATGGGCAAGCTG | CTGTAGTCTTCGGAATGGACGTGCC | |

**References**

[1] S. Chen, Y. Zhou, Y. Chen, J. Gu, *Bioinformatics* **2018**, *34* (17), i884, <https://doi.org/10.1093/bioinformatics/bty560>.

[2] I. Prada-Luengo, A. Krogh, L. Maretty, B. Regenberg, *BMC Bioinformatics* **2019**, *20* (1), 663, <https://doi.org/10.1186/s12859-019-3160-3>.

[3] Y. Wang, M. Wang, M. N. Djekidel, H. Chen, D. Liu, F. W. Alt, Y. Zhang, *Nature* **2021**, *599* (7884), 308, <https://doi.org/10.1038/s41586-021-04009-w>.

[4] J. Wang, J. Qian, Y. Hu, X. Kong, H. Chen, Q. Shi, L. Jiang, C. Wu, W. Zou, Y. Chen, J. Xu, J. Y. Fang, *Nat Commun* **2014**, *5*, 4735, <https://doi.org/10.1038/ncomms5735>.

[5] Z. Li, C. Zhang, C. Li, J. Zhou, X. Xu, X. Peng, X. Zhou, *PLoS Pathog* **2020**, *16* (7), e1008774, <https://doi.org/10.1371/journal.ppat.1008774>.

[6] T. Yang, F. Geng, X. Tang, Z. Yu, Y. Liu, B. Song, Z. Tang, B. Wang, B. Ye, D. Yu, S. Zhang, *MedComm (2020)* **2024**, *5* (7), e625, <https://doi.org/10.1002/mco2.625>.

[7] F. Geng, J. Chen, B. Song, Z. Tang, X. Li, S. Zhang, T. Yang, Y. Liu, W. Mo, Y. Zhang, C. Sun, L. Tan, W. Tu, D. Yu, J. Cao, S. Zhang, *Cell Mol Immunol* **2024**, <https://doi.org/10.1038/s41423-024-01185-3>.

[8] F. Geng, L. Zhong, T. Yang, J. Chen, P. Yang, F. Jiang, T. Yan, B. Song, Z. Yu, D. Yu, J. Zhang, J. Cao, S. Zhang, *Adv Sci (Weinh)* **2024**, e2306253, <https://doi.org/10.1002/advs.202306253>.

[9] J. Luo, X. Zhou, X. Ge, P. Liu, J. Cao, X. Lu, Y. Ling, S. Zhang, *Cancer Sci* **2013**, *104* (11), 1544, <https://doi.org/10.1111/cas.12248>.
